# Functional selectivity of EM-2 analogs at the mu-opioid receptor

**DOI:** 10.3389/fphar.2023.1133961

**Published:** 2023-02-24

**Authors:** Justyna Piekielna-Ciesielska, Davide Malfacini, Francine Medjiofack Djeujo, Chantal Marconato, Karol Wtorek, Girolamo Calo’, Anna Janecka

**Affiliations:** ^1^ Department of Biomolecular Chemistry, Medical University of Lodz, Lodz, Poland; ^2^ Department of Pharmaceutical and Pharmacological Sciences, Section of Pharmacology, University of Padova, Padova, Italy

**Keywords:** opioid receptors, G protein, β-arrestin 2, endomorphin-2 analogs, calcium mobilization assay, BRET assay, bias factor

## Abstract

The mu opioid receptor agonists are the most efficacious pain controlling agents but their use is accompanied by severe side effects. More recent developments indicate that some ligands can differentially activate receptor downstream pathways, possibly allowing for dissociation of analgesia mediated through the G protein from the opioid-related side effects mediated by β-arrestin pathway. In an effort to identify such biased ligands, here we present a series of thirteen endomorphin-2 (EM-2) analogs with modifications in positions 1, 2, and/or 3. All obtained analogs behaved as mu receptor selective agonists in calcium mobilization assay carried out on cells expressing opioid receptors and chimeric G proteins. A Bioluminescence Resonance Energy Transfer (BRET) approach was employed to determine the ability of analogs to promote the interaction of the mu opioid receptor with G protein or β-arrestin 2. Nearly half of the developed analogs showed strong bias towards G protein, in addition four compounds were nearly inactive towards β-arrestin 2 recruitment while blocking the propensity of EM-2 to evoke mu-β-arrestin 2 interaction. The data presented here contribute to our understanding of EM-2 interaction with the mu opioid receptor and of the transductional propagation of the signal. In addition, the generation of potent and selective mu receptor agonists strongly biased towards G protein provides the scientific community with novel tools to investigate the *in vivo* consequences of biased agonism at this receptor.

## 1 Introduction

Opioid receptors (mu, delta, and kappa) belong to the family of the G protein-coupled receptors (GPCRs) and are responsible for pain perception and mediation of other effects of opioids. They are targeted by endogenous ligands of peptide structure (endomorphins, enkephalins, dynorphins) as well as opiate alkaloids, such as morphine, which is one of the most clinically effective analgesics. Among the three types of opioid receptors, the mu receptor was identified as the one essential for the pain-relieving effects but also responsible for a number of undesired side effects, including sedation, respiratory depression, inhibition of gastrointestinal transit, and also development of tolerance and physical dependence (Benyamin et al., 2008). Moreover the misuse and abuse of opioid analgesics led in the last decades to the so called opioid epidemic (Blanco et al., 2022) making urgent the identification of novel opioid drugs with lower abuse liability and/or safer profile (Varga et al., 2021).

Opioid receptors are integral membrane proteins. Their activation leads to the initiation of internal signal transduction pathways and cellular responses. GPCRs transduce signals through coupling to heterotrimeric G proteins (Ahn et al., 2004). Phosphorylation of the agonist-occupied receptor by G protein-coupled receptor kinases (GRKs) leads to β-arrestin recruitment, followed by inhibition of further receptor-G protein interactions by receptor desensitization or internalization (Goodman et al., 1996; Dang et al., 2011; Violin et al., 2014). It has also been discovered that β-arrestin can independently transduce some intracellular signaling pathways and therefore GPCRs may activate distinct biochemical responses depending on the recruitment of either G proteins or β-arrestins (Ma & Pei, 2007; Defea, 2008; Chen & Tesmer, 2022). Experiments with β-arrestin knockout mice showed that in such animals morphine caused enhanced and longer-lasting antinociception with reduced side effects as compared with wild type mice (Bohn et al., 1999; Bohn et al., 2002; Lamberts and Traynor, 2013).

On the other hand, a peripherally restricted mu receptor agonist, loperamide, which is a well-known antidiarrheal agent, significantly reduced colonic propulsion in wild type mice, but was completely inactive in the β-arrestin knockout mice (Raehal et al., 2005).

These and similar data indicate that analgesic effect evoked by mu opioid receptor agonists results mostly from the activation of the receptor and subsequent signaling through the G protein, while some side effects may be induced by the β-arrestin pathway (Manglik et al., 2016).

However, it should be mentioned that recent findings (Gillis et al., 2020a; Azevedo Neto et al., 2020) speak against this view, and rather propose that reduced efficacy is critical for the safer profile displayed by putative mu receptor biased agonists.

The first mu opioid G protein-biased non-peptide ligand, oliceridine (TRV 130), caused limited β-arrestin recruitment but had similar to morphine potency and efficacy at the mu opioid receptor (Chen et al., 2013). In rodents administration of oliceridine at doses equi-analgesic to morphine, produced strong antinociceptive effect with reduced influence on gastrointestinal transit and respiratory system (Dewire et al., 2013). Following phase III clinical trials, in 2020 oliceridine was approved for short-term intravenous use in hospitals and other controlled clinical settings in the United States under the brand name Olinvyk (FDA, 2020). It is highly effective for the treatment of moderate to severe acute pain in adults, as the lack of efficacy was observed in less than 5% of patients. Nevertheless, 64% of patients experienced some adverse effects such as vomiting, nausea, headache, or constipation (Bergese et al., 2019). Therefore, the efforts to develop novel analgesics with improved side effect profiles is continued.

Opioid peptides might be an alternative to morphine-based drugs, as they have high potency, exquisite selectivity and low toxicity (Czapla et al., 2000; Wilson et al., 2000). In the search for biased ligands of peptide structure, we turned our attention to endogenous mu opioid receptor ligands, endomorphins (EM-1, Tyr-Pro-Trp-Phe-NH_2_, and EM-2, Tyr-Pro-Phe-Phe-NH_2_) (Zadina et al., 1997). Similarly to morphine, EMs activate both G protein and β-arrestin pathways (Lamberts and Traynor. 2013). In this study we synthesized a series EM-2 analogs with modifications in positions 1, 2, and 3, designed to enhance their enzymatic stability, bioavailability and functional selectivity as compared with the parent compound.

The affinity of all ligands at the mu, delta, and kappa opioid receptors was evaluated in binding assays and their functional activity in a calcium mobilization assay performed in cells expressing chimeric G proteins, and with a Bioluminescence Resonance Energy Transfer (BRET) assay capable to assess analogs propensity to promote mu-G protein or mu-β-arrestin 2 interaction. Enzymatic degradation and lipophilicity studies were carried out to select the most stable peptides with improved bioavailability as compared with EM-2. Here, we present a series of enzymatically stable, membrane penetrant, mu receptor selective agonists with different degrees of bias towards G protein.

## 2 Materials and methods

### 2.1 General methods

Reagents used in the experiments were obtained from Sigma Aldrich, unless otherwise stated. Protected amino acids were purchased from Trimen Co., (Lodz, Poland). Concentrated solutions of peptides were made in ultrapure water (1 mM) and kept at—20°C until use.

Analytical and semi-preparative RP HPLC was performed using Waters Breeze instrument (Milford, MA, United States) with dual absorbance detector (Waters 2,487) on a Vydac C_18_ column 5 μm, 4.6 × 250 mm, flow rate 1 mL/min, 50 min linear gradient, and a Vydac C_18_ column 10 μm, 22 × 250 mm, flow rate 2 mL/min, 20 min linear gradient, respectively, from water/0.1% (v/v) TFA to 80% acetonitrile/20% water/0.1% (v/v) TFA. ESI-MS spectra were obtained on an FTICR (Fourier transform ion cyclotron resonance) Apex-Qe Ultra 7 T mass spectrometer (Bruker Daltonics, Bremen, Germany) equipped with standard ESI source. The instrument was operated in the positive-ion mode and calibrated with the Tunemix™ mixture (Agilent Technologies, Palo Alto, CA, United States). Solutions of peptides were introduced at a flow rate of 3 μL/min.

### 2.2 Peptide synthesis

Peptides were synthesized by Trimen Co., (Lodz, Poland). The purity of final products was in each case greater than 95%, as assessed by analytical RP-HPLC. Identity of the synthesized peptides was confirmed by MC-MS (Supplementary Figure S1).

### 2.3 Metabolic stability

Enzymatic degradation of EM-2 and the analogs was performed measuring their hydrolysis rates in the presence of rat brain homogenate. The homogenate was prepared prior to the experiment. Briefly, rat brains were homogenized in 20 volumes of Tris–HCl (50 mM, pH 7.4) using a Polytron and the obtained aliquots were stored at −80°C. In the experiment, aliquotes of brain homogenate (100 μL, 10 mg protein/mL) were incubated with 100 µL of a peptide (0.5 mM) over 0, 7.5; 15, 22.5; 30, and 60 min at 37°C, in a final volume of 200 µL. After the incubation the degradation reaction was stopped by placing the tube on ice and acidifying the content with 20 µL of HCl (1 M). The aliquots were centrifuged at 20,000 g for 10 min at 4°C. The supernatants were collected and filtered using Millex-GV syringe filters (Millipore). Then the analysis by HPLC on a Vydac C_18_ column (5 μm, 4.6 × 250 mm), using the solvent system of 0.1% TFA in water (A) and 80% acetonitrile in water containing 0.1% TFA (B) and a linear gradient of 0%–100% B over 25 min, was performed. Three independent experiments for each assay were carried out in duplicate. The rate constants of degradation (k) were obtained by the least square linear regression analysis of logarithmic peak areas [ln (A/A_D_)], where A—amount of remained peptide, A_D_—initial amount of peptide *versus* time. Degradation half-lives (t_1/2_) were calculated from the rate constants as ln2/k.

### 2.4 n-Octanol/water partition coefficient

Determination of log *P* was performed according to the method described by (Liu et al., 2006). Peptides were dissolved in 0.05 M HEPES buffer in 0.1 M NaCl, pH 7.4, then an equal volume of n-octanol was added and mixtures were vortexed for 2 min. Samples were centrifuged at 4,000 rpm for 1 min. After separation, aqueous and octanol phases were used to quantify peptide content by RP-HPLC.

Octanol phase was lyophilized and reconstituted in 80% acetonitrile in water containing 0.1% TFA before RP-HPLC. All n-octanol/buffer distribution studies were performed in triplicate.

### 2.5 Calcium mobilization functional assay

Stock solutions (10 mM) of the peptides were prepared in 5% DMSO in bidistilled water and kept at −20°C Serial dilutions were carried out in HBSS/HEPES buffer (20 mM, containing 0.02% bovine serum albumin fraction V). Calcium mobilization assay was performed using the same method as previously described (Camarda and Calo, 2013). CHO cells with stable co-expression of human mu or kappa receptors and the C-terminally modified Gα_qi5_ and CHO cells with co-expression of the delta-opioid receptor and the Gα_qG66Di5_ protein were used. Dulbecco’s MEM/HAMS F12 (1:1) medium supplemented with 10% fetal bovine serum, penicillin (100 IU/mL), streptomycin (100 μg/mL), L-glutammine (2 mM), fungizone (1 μg/mL), geneticin (G418; 200 μg/mL) and hygromycin B (100 μg/mL) was used for cell culture. Cells were seeded at a density of 50,000 cells/well into 96-well black, clear-bottom plates and kept in the incubator at 37°C in 5% CO_2_/humidified air. After 24 h the cell growth medium was aspired and loading medium, supplemented with probenecid (2.5 mM), calcium sensitive fluorescent dye Fluo-4 AM (3 µM), pluronic acid (0.01%) and HEPES (20 mM), was added. Then the plates were placed in the incubator again. After 30 min the loading solution was aspirated and 100 µL/well of assay buffer (HBSS supplemented with 20 mM HEPES, 2.5 mM probenecid, and 500 µM Brilliant Black) was added. Next, both plates (cell culture and compound plate) were placed in the FlexStation II reader (Molecular Device, Union City, CA 94587, United States), the on-line additions were carried out in a volume of 50 µL/well and the fluorescence changes were measured. Ligand efficacies, expressed as the intrinsic activity (α), were calculated as the E_max_ ratio of the tested compound and the standard agonist. At least three independent experiments for each assay were carried out in duplicate.

Curve fittings were performed using Graph Pad PRISM 5.0 (GraphPad Software Inc., San Diego, United States). Data have been statistically analyzed with one way ANOVA followed by the Dunnett’s test for multiple comparisons; *p* values < 0.05 were considered significant.

### 2.6 Bioluminescence Resonance Energy Transfer (BRET) receptor-transducer interaction assay

*In vitro* pharmacological profile of mu-opioid receptors ligands was evaluated by testing receptor interaction with G protein and β-arrestin 2 with a BRET interaction assay, as previously reported by (Molinari et al., 2010). SH-SY5Y human neuroblastoma cells stably co-expressing two pairs of fusion proteins were developed employing a pantropic retroviral expression system (Clontech) as previously described (Molinari et al., 2010). RLuc-tethered human mu receptor fusion protein and the bovine Gβ1 and the human β-arrestin 2 N-terminal-tagged with RGFP (Prolume, Pinetop, United States) were generated as previously detailed (Molinari et al., 2010).

SH-SY5Y cells stably co-expressing the fusoproteins mu-RLuc and Gβ1-RGFP or mu-RLuc and β-arrestin 2-RGFP were grown in DMEM/F12 (1:1) medium supplemented with 10% FBS, 2 mM L-Glutamine, 100 μg/mL hygromycin B, 400 μg/mL G418, 100 units/mL penicillin G, 100 μg/mL streptomycin and 1 μg/mL Fungizone at 37°C in a humidified atmosphere with of 5% CO_2_.

*Mu-G protein interaction*—Enriched plasma membrane samples from mu-RLuc/Gβ1-RGFP expressing cells for receptor-G protein interaction assay were prepared by differential centrifugation as described previously (Vachon et al., 1987). Total protein in membrane preparations was determined by colorimetric method with the Quantum Protein-BCA kit (EuroClone, Pero (MI), IT) and measured using the multiplate reader Victor Nivo (PerkinElmer, Walthman, MA, United States). White opaque 96 wells microplates (PerkinElmer, Walthman, MA, United States) were used to carry out BRET assays. Cell membranes were thawed and resuspended in PBS supplemented with 0.02% BSA before the assay, and an amount of 5 μg of total protein was dispensed in each 96 well. All experiments were carried out at room temperature.

*Mu- β-arrestin 2 interaction—*Living SH-SY5Y cells expressing mu-RLuc and β-arrestin 2-RGFP were seeded at a cell density of 100,000 cells/well 24 h prior to the test. On the day of the experiment, medium was discarded, and cells were washed with PBS supplemented with 0.5 mM MgCl_2_, 0.9 mM CaCl_2_. Cells were subsequently incubated with 2 μM Prolume Purple Coelenterazine (NanoLight Technology, White Mountain, AZ; United States) for 10 min before bioluminescence reading.

In agonism experiments, ligands were added and incubated for 5 min before microplate reading. The Victor 2030 luminometer (PerkinElmer, Waltham, MA, United States) was employed to measure counts per second (CPS), emitted lights were selected using 405 (10) and 510 (30) bandpass filters for Rluc and RGFP, respectively. BRET ratios were computed as follow:

$${\left(\frac{{RGFP}_{CPS}}{{RLuc}_{CPS}}\right)}_{ligand}-{\left(\frac{{RGFP}_{CPS}}{{RLuc}_{CPS}}\right)}_{vehicle}$$

Effects of agonists were expressed normalized to EM-2 (equal to 1.00) following vehicle subtraction.

In antagonism experiments, a 10 min preincubation with vehicle or fixed concentrations of ligand preceded the injection of increasing concentrations of EM-2. BRET ratios were then measured during the subsequent 15 min.

*RLuc interference*—These experiments were performed on cell membranes prepared and as above described. Because several ligands are described to interact with RLuc or to generate unspecific luminescence artifacts (Auld et al., 2008), the eventual ligand-RLuc light alteration was quantified. SH-SY5Y cells used expressed the mu-RLuc and the β-arrestin 2-RGFP fusoproteins. During the membrane preparation routine, cytosolic β-arrestin 2-RGFP is washed out, but not the mu-RLuc, allowing for precise quantification of RLuc light emission alteration. We considered 15% alteration of vehicle RLuc-CNTZ emission as the threshold for excluding the compound’s concentration from subsequent experiments.

### 2.7 Data analysis and terminology

The pharmacological terminology employed is consistent with the International Union of Basic and Clinical Pharmacology (IUPHAR) recommendations (Neubig et al., 2003). Concentration-response curves to agonists were fitted to the four-parameter logistic non-linear regression model as follows:

$$Effect=Basal+\frac{E_{\max}-Basal}{1+10^{\left(\mathrm{Log}{EC}_{50}-\mathrm{Log}\left[ligand\right]\right)\times HillSlope}}$$

Curve fitting was performed using PRISM 8.0 (GraphPad Software Inc., San Diego, CA).

Bias factors, a measure of the differences in agonist propensity to foster mu to G protein or β-arrestin 2 interactions, were calculated considering EM-2 as standard ligand (ligand benchmark-bias). For this analysis, the E_max_ and EC_50_ of the agonist were derived using a 3-parameter logistic model as previously described (Malfacini et al., 2015). The following formula was used for calculating agonist bias factors in log_10_ units:

bias factor=logEmaxEC50ligEmaxEC50EM−2G prot.−logEmaxEC50ligEmaxEC50EM−2β−arr.

The pharmacological terminology and computations related to biased agonism were consistent with IUPHAR recommendations (Kolb et al., 2022). Bias factors were considered significantly different from the reference ligand when a ligand’s CL_95%_ didn’t include zero.

Antagonist potencies (pK_b_) were computed applying the following equation: pK_b_ = (log (CR − 1)) − log [Anta] with CR as the ratio between agonist potency (molar) in the presence and absence of antagonist and [Anta] is the molar concentration of antagonist. pK_b_ represents the concentration of antagonist that occupies half of the receptor population at equilibrium, expressed in molar units.

All data are expressed as mean ± SEM of n experiments and were analyzed statistically using one-way analysis of variance followed by Dunnett’s test for multiple comparisons. Potency values and bias factors are expressed as mean (CL_95%_).

## 3 Results

### 3.1 Peptide design

Seven pairs of EM-2 analogs were synthesized. Peptides in each pair differed by the amino acid in position 1 (Tyr or 2′,6′-dimethyltyrosine, Dmt). It was shown in the past that introduction of Dmt at the N-terminus of various opioid peptides resulted in elevation of mu opioid receptor affinities (Okada et al., 2003). Such increases can be explained by additional interactions of the two methyl groups on the aromatic ring of Dmt with the binding pocket of the receptor. The methyl groups may also enhance peptide lipophilicity, enabling permeability of peptides through biological membranes.

Other modifications, designed to enforce enzymatic stability of analogs, included replacement of Pro in position 2 by its six-membered surrogates, piperidine-2- or 3-carboxylic acids [(*R*)-Pip or (*R*)-Nip, respectively] or β-alanines, (*R*)-β^2^-Ala or (*R*)-β^3^-Ala. Finally, naphthylalanines, (*R*)-β^2^-1-Nal or (*R*)-β^3^-1-Nal were used to substitute Phe in position 3. The amino acid sequences of the new analogs are shown in Table 1.

**TABLE 1:** Enzymatic stability and lipophilicity (log *P*) of EM-2 analogs.

No.	Sequence	Purity	100 x *k* (per min)	t_1/2_ (min)	Log *P*
EM-2	Tyr-Pro-Phe-Phe-NH_2_	98	9.27 ± 0.42	7.4 ± 0.5	1.19
**1**	**Dmt**-Pro-Phe-Phe-NH_2_	97	3.31 ± 0.12	20.8 ± 1.2	2.16
**2**	Tyr**-(*R*)-Pip**-Phe-Phe-NH_2_	97	0.57 ± 0.08	121.2 ± 3.1	2.30
**3**	**Dmt-(*R*)-Pip**-Phe-Phe-NH_2_	96	0.73 ± 0.07	94.5 ± 2.8	2.49
**4**	Tyr**-(*R*)-Nip-**Phe-Phe-NH_2_	98	1.05 ± 0.08	65.7 ± 2.4	1.68
**5**	**Dmt-(*R*)-Nip**-Phe-Phe-NH_2_	98	0.77 ± 0.06	89.6 ± 3.0	1.80
**6**	Tyr**-(*R*)-β** ^**2**^ **-Ala**-Phe-Phe-NH_2_	96	1.03 ± 0.07	66.9 ± 2.7	1.75
**7**	**Dmt-(*R*)-β** ^**2**^ **-Ala**-Phe-Phe-NH_2_	97	0.51 ± 0.03	135.3 ± 4.3	2.33
**8**	Tyr**-(*R*)-β** ^**3**^ **-Ala**-Phe-Phe-NH_2_	97	0.82 ± 0.04	84.2 ± 3.9	1.71
**9**	**Dmt-(*R*)-β** ^**3**^ **-Ala**-Phe-Phe-NH_2_	96	1.90 ± 0.04	36.3 ± 2.8	2.09
**10**	Tyr-Pro**-(*R*)-β** ^**2**^ **-1-Nal**-Phe-NH_2_	97	0.41 ± 0.02	168.3 ± 5.5	2.50
**11**	**Dmt**-Pro**-(*R*)-β** ^**2**^ **-1-Nal**-Phe-NH_2_	96	0.56 ± 0.02	123.2 ± 5.9	2.71
**12**	Tyr-Pro**-(*R*)-β** ^**3**^ **-1-Nal**-Phe-NH_2_	96	1.28 ± 0.05	53.9 ± 4.1	3.01
**13**	**Dmt**-Pro**-(*R*)-β** ^**3**^ **-1-Nal**-Phe-NH_2_	98	1.69 ± 0.07	40.8 ± 4.0	3.19

### 3.2 Enzymatic stability of EM-2 analogs

The resistance of the analogs to enzymatic degradation by proteolytic enzymes present in rat brain homogenate was evaluated in comparison with EM-2. The selected time period (60 min of incubation of peptides with the homogenate) was sufficient to observe differences between EM-2 and its analogs. The degradation of EM-2 was rapid (t_1/2_ = 7.4 min), whereas modification of its structure by incorporation of Dmt^1^ resulted in noticeably increased resistance to hydrolysis by proteolytic enzymes (peptide **1**, t_1/2_ = 20.8 min). Additional incorporation of unnatural amino acids in position 2 or 3 led to further enhancement of enzymatic stability of the obtained analogs. The most stable analog **10** was characterized by t_1/2_ = 168 min (Table 1).

### 3.3 n-Octanol/water partition coefficient

Lipophilicity is an important parameter influencing the ability of a peptide to cross biological membranes by passive diffusion. Lipophilicity can be expressed by log *P,* which is the ratio of a peptide concentration in two immiscible solvents, usually *n*-octanol and water.

Log *P* values obtained for EM-2 and the new analogs are presented in Table 1. All analogs showed increased lipophilicity in comparison with the parent peptide, EM-2. As expected, lipophilicity of compounds with Dmt^1^ was in all cases higher than that of the corresponding peptides with Tyr^1^. The highest log *P* values for analogs modified in position 2 were found for the pair **2** and **3,** incorporating (*R*)-Pip (2.30 and 2.49, respectively). Among peptides modified in position 3, analogs **12** and **13**, containing (*R*)-β^3^-1-Nal showed the highest lipophilicity (3.01 and 3.16 respectively). The values of lipophilicity for other analogs ranged between 1.68–2.71 which is optimal for effective blood-brain barrier permeability.

### 3.4 Radioligand binding assay

The affinities of EM-2 and analogs **1–13** for all three opioid receptors were determined by binding experiments performed on commercial membranes of CHO cells transfected with human recombinant opioid receptors, using [^3^H]DAMGO, [^3^H]deltorphin-2 and [^3^H]U-69593 as radioligands for mu, delta and kappa receptor, respectively (Table 2). All new analogs displayed sub-nM or nM mu affinity, which for compounds **1**, **4**, **5**, **7**, **8**, **9** was higher than the affinity of EM-2 used as a reference opioid ligand. The peptides had no substantial affinity for delta and kappa receptors, being therefore highly mu-selective. The only exception was compound **3** which showed moderate delta and kappa affinity with K_i_ values of 37 and 72 nM, respectively.

**TABLE 2:** Opioid receptor binding of EM-2 analogs.

No.	K_i_ ^a^ ± SEM [nM]		
	mu	Delta	Kappa
EM-2	1.97 ± 0.21	>1,000	>1,000
**1**	0.54 ± 0.03	560 ± 41	830 ± 60
**2**	17.72 ± 1.14	682 ± 33	>1,000
**3**	3.92 ± 0.50	37 ± 5	72 ± 6
**4**	0.85 ± 0.17	>1,000	>1,000
**5**	0.14 ± 0.06	589 ± 28	567 ± 39
**6**	46.80 ± 5.18	>1,000	>1,000
**7**	0.31 ± 0.09	571 ± 55	807 ± 46
**8**	0.44 ± 0.10	>1,000	>1,000
**9**	0.14 ± 0.07	286 ± 18	890 ± 42
**10**	34.88 ± 3.9	>1,000	>1,000
**11**	9.54 ± 0.94	675 ± 56	>1,000
**12**	32.14 ± 4.05	>1,000	>1,000
**13**	2.75 ± 0.69	722 ± 49	915 ± 67

^a^ Displacement of [^3^H]DAMGO (mu-selective), [^3^H]deltorphin-2 (delta-selective) and [^3^H]U-69593 (kappa-selective) from human opioid receptor membrane binding sites. All values are expressed as mean ± SEM, n ≥ 3.

### 3.5 Calcium mobilization functional assay

**FIGURE 1:**
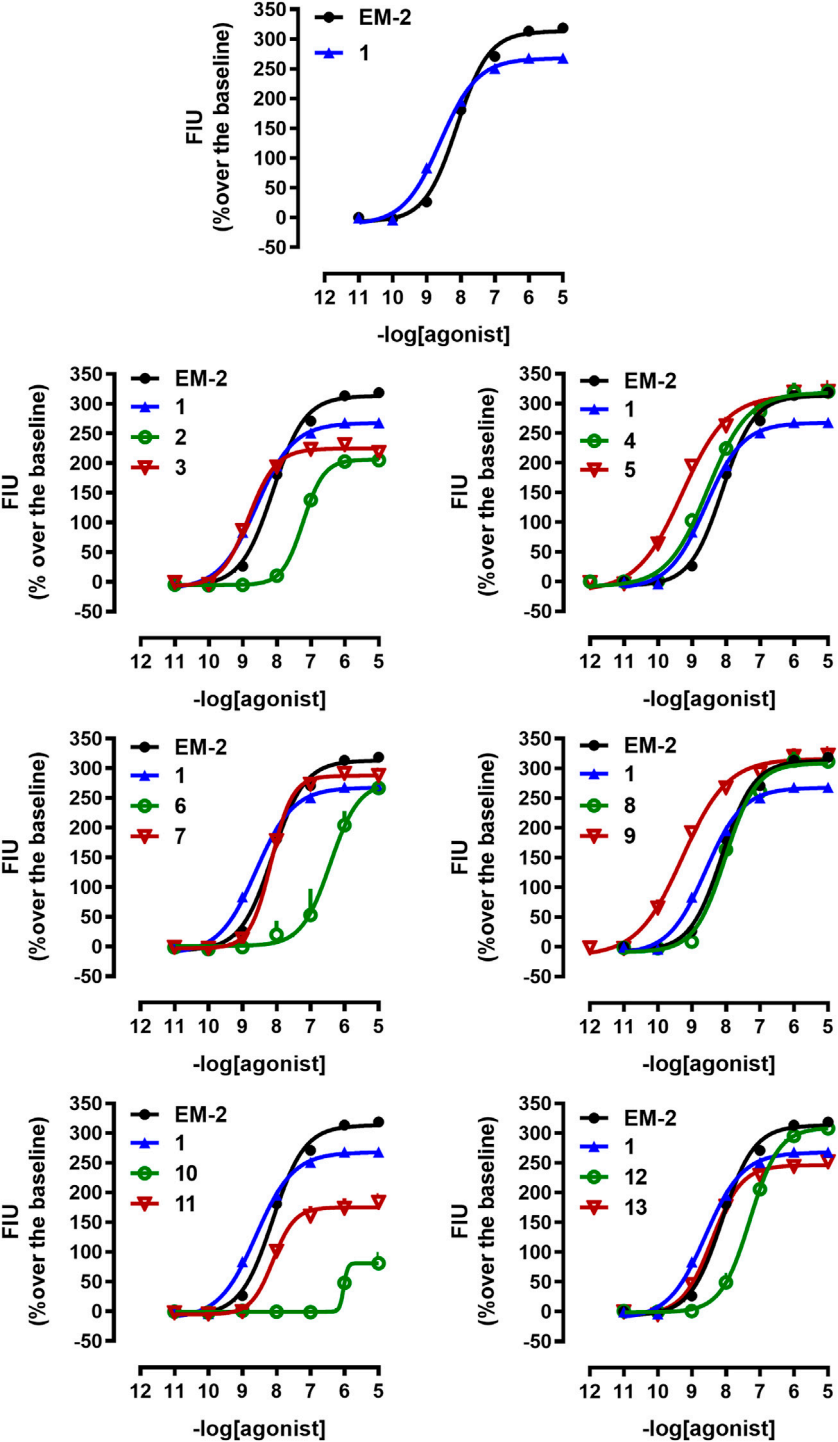
Calcium mobilization experiments at mu receptor. Concentration response curves to standard (**EM-2**) and tested compounds (**1–13**) in calcium mobilization experiments performed in CHO_mu_ cells stably expressing the Gα_qi5_ protein. Data are the mean ± SEM of at least 5 separate experiments performed in duplicate.

All tested compounds turned out to be selective agonists of the mu opioid receptor. Analogs containing Dmt in position 1 showed generally higher potency than the corresponding compounds with Tyr. Substitution of Tyr^1^ by Dmt in EM-2 resulted in a 3-fold higher potency, but slightly decreased E_max_ (analog **1**). The highest potency was observed for analogs **5** and **9**, with Dmt in position 1 and (*R*)-Nip or (*R*)-β^3^-Ala in position 2, respectively (Table 3; Figure 1).

**TABLE 3:** Effects of reference agonists and novel peptides at human recombinant opioid receptors in a calcium mobilization assay performed in cells expressing chimeric G proteins.

Peptide	mu		Delta		Kappa	
	pEC_50_ (CL_95%_)	α±SEM	pEC_50_ (CL_95%_)	α± SEM	pEC_50_ (CL_95%_)	α± SEM
EM-2	8.08 (7.99–8.15)	1.00	inactive^a^		inactive^a^	
DPDPE	inactive^b^		7.27 (7.03–7.51)	1.00	Inactive^b^	
dynorphin A	6.67^b^ (6.17–7.17)	0.83 ± 0.10^b^	7.73^b^ (7.46–8.00)	0.99 ± 0.04^b^	8.86 (8.50–9.22)	1.00
**1**	8.60 (8.51–8.69)	0.85 ± 0.02	inactive		Inactive	
**2**	7.23 (7.11–7.36)	0.64 ± 0.04	inactive		inactive	
**3**	8.82 (8.70–8.93)	0.70 ± 0.03	6.83 (6.56–7.10)	0.56 ± 0.05	inactive	
**4**	8.57 (8.36–8.78)	0.99 ± 0.04	inactive		inactive	
**5**	9.50 (9.15–9.85)	1.00 ± 0.02	inactive		inactive	
**6**	7.52 (6.84–8.20)	0.88 ± 0.06	inactive		inactive	
**7**	9.16 (9.09–9.22)	0.95 ± 0.03	inactive		inactive	
**8**	9.00 (8.73–9.28)	0.99 ± 0.02	inactive		inactive	
**9**	9.34 (9.05–9.63)	1.00 ± 0.05	inactive		inactive	
**10**	6.91 (5.85–6.57)	0.25 ± 0.06	inactive		inactive	
**11**	8.01 (7.85–8.18)	0.57 ± 0.05	inactive		inactive	
**12**	7.32 (7.07–7.57)	0.97 ± 0.02	inactive		inactive	
**13**	8.42 (8.19–8.65)	0.77 ± 0.02	inactive		inactive	

^a^ Inactive means that the compound was inactive up to 10 µM.

^b^ Data from Perlikowska et al., 2014.

With regard to delta agonism, only compound **3** evoked calcium signaling responses, but with reduced maximal effects (Table 3; Supplementary Figure S2).

As for potency at kappa opioid receptor, none of the tested peptides was active (Table 3; Supplementary Figure S3).

### 3.6 BRET receptor-transducer interaction assay

**FIGURE 2:**
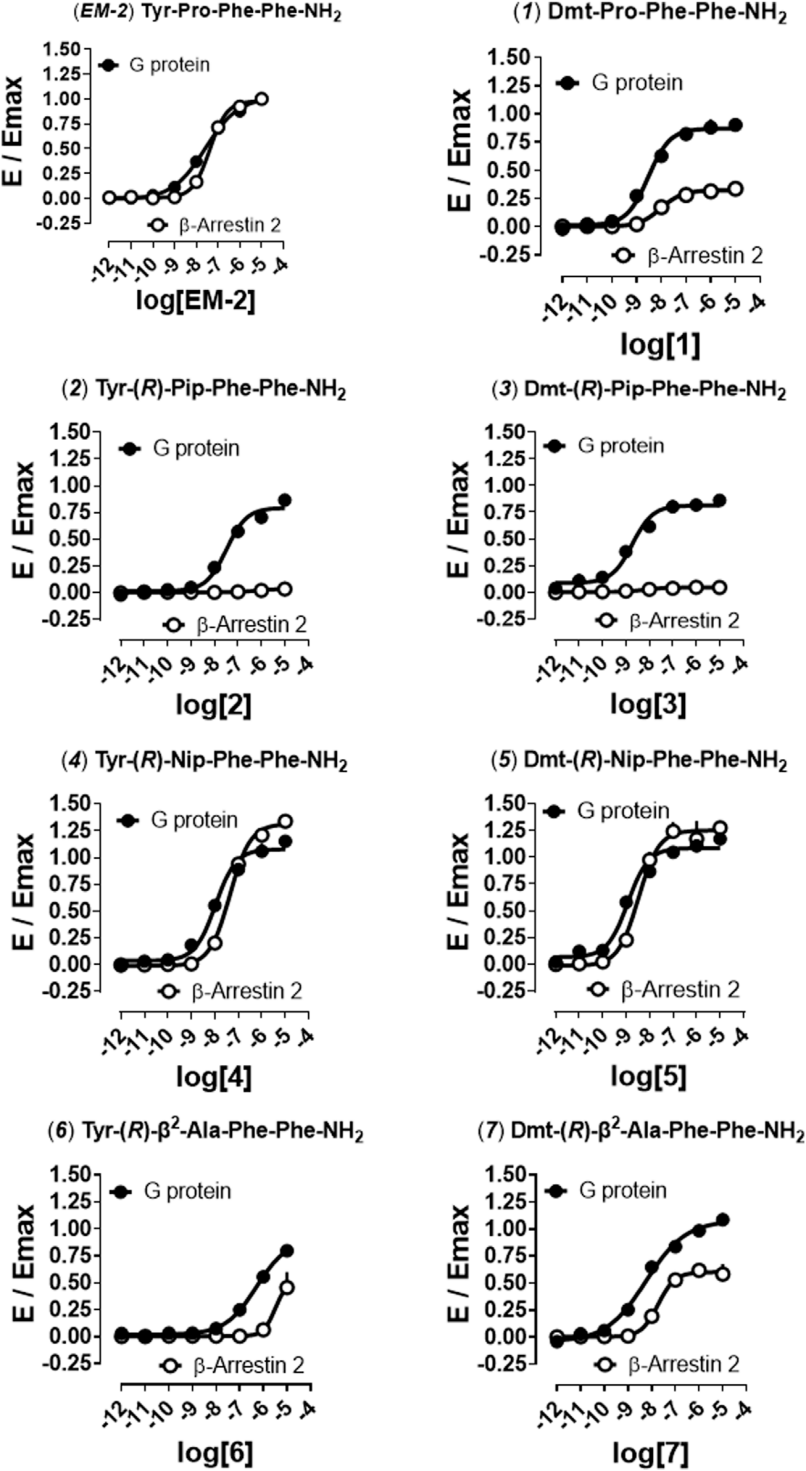
Mu receptor-transducer interactions (*EM-2, 1, 2–7*). Effects of EM-2, **2**, **4**, **6**, and corresponding Dmt^1^ derivatives (**1**, **3**, **5**, **7**) in mu -G protein and -β-arrestin 2 BRET interaction assays. Data are mean + SEM of at least four independent experiments performed in duplicate. Corresponding bias plots are reported in Supplementary Figure S5.

**FIGURE 3:**
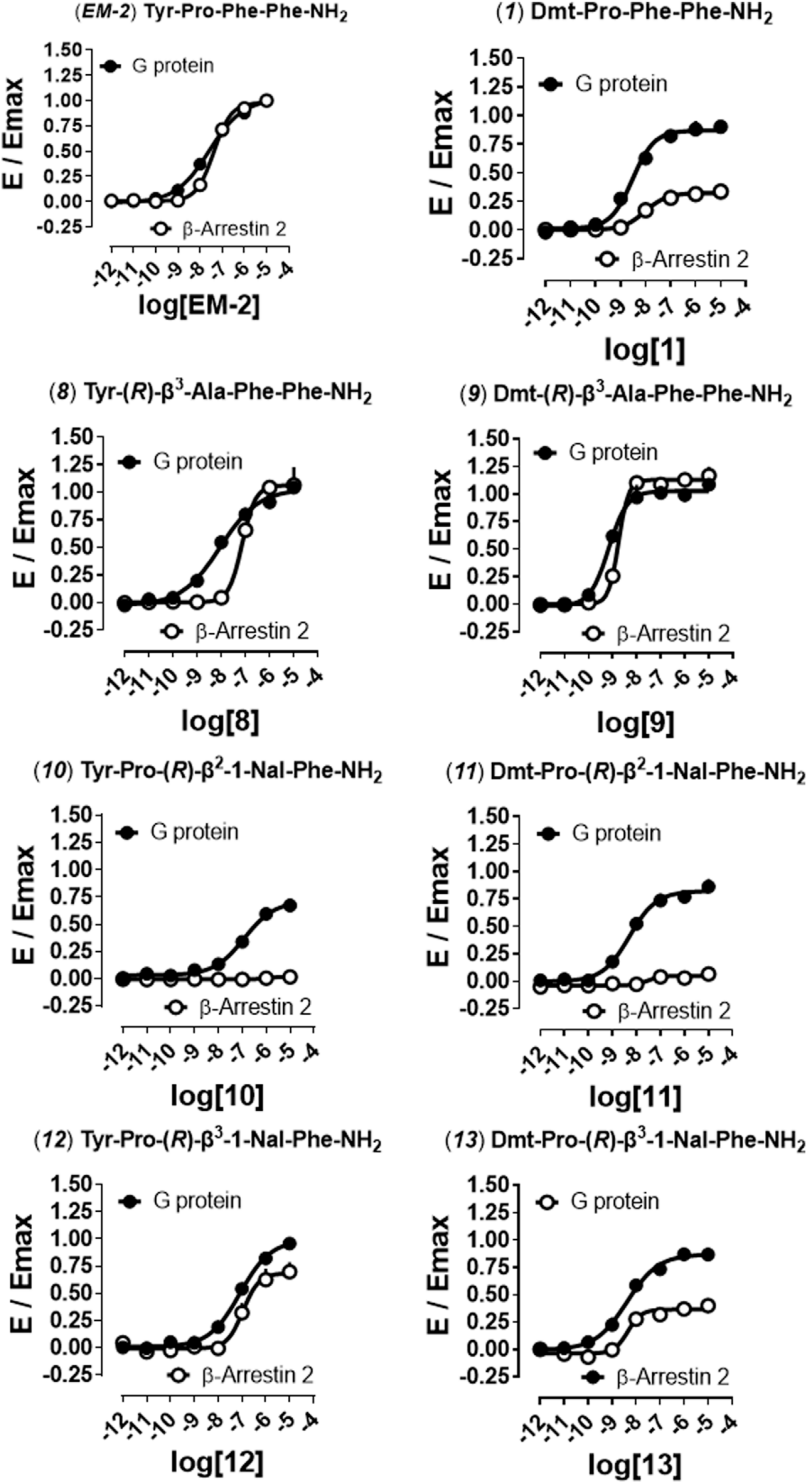
Mu receptor-transducer interactions **(EM-2, 1, 8–9).** Effects of EM-2 and analog **1** reported for comparison to the activities of analogs **8**, **10**, and **12**, and corresponding Dmt^1^ derivatives (**9**, **11**, **13**) in mu-G protein and -β-arrestin 2 BRET interaction assays. Data are mean + SEM of at least four independent experiments performed in duplicate. Corresponding bias plots are reported in Supplementary Figure S5.

In order to distinguish between the activity of the new molecules towards mu-G protein and mu-β-arrestin 2 couplings a BRET receptor-transducer interaction assay was performed. First, it was shown that none of the EM-2 analogs altered the light emitted by Rluc (Supplementary Figure S4). Then, the naturally occurring peptide EM-2 and all thirteen derivatives were assayed for their propensity to foster mu receptor interaction with G protein and β-arrestin 2 (Figures 2, 3). Obtained EM-2 potencies were moderate and similar for G protein and β-arrestin 2 (Figure 2). The substitution of Tyr^1^ by Dmt increased potency of about 6-fold at both transducers, while slightly diminishing G protein efficacy and greatly reducing β-arrestin 2 efficacy (α = 0.3) (analog **1**, Figure 2). Replacement of Pro^2^ by (*R*)-Pip (analog **2**) produced a slight decrease of G protein potency and efficacy, while mu-β-arrestin 2 recruitment was almost abolished (Figure 2). The substitution of Tyr^1^ with Dmt (analog **3**) increased potency about 10-fold without altering efficacy at G protein or modifying the activity at β-arrestin 2 (Figure 2). Importantly, although precise estimation of bias factors could not be carried out due to their very low efficacy at β-arrestin 2, analogs **2** and **3** displayed strong bias toward G protein (Figure S5). The introduction of (*R*)-Nip instead of Pro^2^ (analog **4**) slightly increased potency and efficacy at both transducers (Figure 2); additionally, the substitution of Tyr^1^ with Dmt (analog **5**) produced further increase in potency (Figure 2). Analogs **six to nine** had (*R*)-β^2^-Ala or (*R*)-β^3^-Ala in position 2 (Figures 2, 3). Analog **6** displayed the largest loss of potency both at G protein (27-fold) and β-arrestin 2 (approximately 40-fold) (Figure 2). The introduction of Dmt^1^ (analog **7**) reversed this effect at both transducers (Figure 2). Substitution of Pro^2^ with (*R*)-β^3^-Ala (analog **8**) did not change pharmacological parameters (Figure 3), while introduction of Dmt^1^ (analog **9**) generated the greatest gain of potency (20-fold) at both transducers (Figure 3). Finally, β^2^-1-Nal and β^3^-1-Nal, both in *R*-configuration, were employed in position 3. The potency of analog **10** was approximately 7-fold lower than that of EM-2 at G protein, while at β-arrestin 2 this analog was inactive (Figure 3). Introduction of Dmt^1^ (analog **11**) strongly reversed the potency loss at G protein (Figure 3). Analogues **10** and **11** bias could not be estimated, because of the lack of activity at β-arrestin 2; however, inspection of bias plots highlights a very strong bias towards G protein (Supplementary Figure S5). Analog **12** displayed similar loss of potency at both transducers (Figure 3), the introduction of Dmt^1^ (analog **13**) reversed this effect (Figure 3).

Agonist pharmacological parameters of the tested analogs are summarized in Table 4.

**TABLE 4:** Pharmacological parameters of EM-2 and its derivatives on mu-G protein and mu-β-arrestin 2 interaction assays.

	G protein		β-Arrestin 2		
	pEC_50_ (CL_95%_)	E_max_±sem	pEC_50_ (CL_95%_)	E_max_±sem	Bias factor (CL_95%_)
**EM-2**	7.72 (7.39–8.06)	1	7.15 (6.92–7.38)	1	0.00
**1**	8.51 (8.31–8.71)	0.90 ± 0.05	7.93 (7.52–8.33)	0.33 ± 0.02	0.48 (0.34–0.62)
**2**	7.42 (7.18–7.65)	0.83 ± 0.06	Inactive		n.d
**3**	8.72 (8.58–8.87)	0.83 ± 0.04	Inactive		n.d
**4**	7.95 (7.77–8.13)	1.11 ± 0.07	7.36 (7.28–7.44)	1.30 ± 0.03	−0.02 (−0.04-0.00)
**5**	8.96 (8.79–9.13)	1.11 ± 0.04	8.44 (8.18–8.71)	1.26 ± 0.08	−0.08 (−0.25-0.10)
**6**	6.38 (6.13–6.64)	0.86 ± 0.05	4.85^a^ (4.38–5.32)	∼1^a^	1.35 (1.15–1.55)
**7**	8.34 (8.11–8.57)	0.98 ± 0.02	7.72 (7.58–7.85)	0.61 ± 0.06	0.67 (0.49–0.84)
**8**	8.13 (7.83–8.43)	0.96 ± 0.04	7.11 (6.90–7.32)	1.12 ± 0.10	0.76 (0.55–0.98)
**9**	9.14 (9.00–9.29)	1.05 ± 0.02	8.67 (8.59–8.76)	1.16 ± 0.05	0.27 (0.16–0.38)
**10**	6.98 (6.84–7.11)	0.68 ± 0.04	Inactive		n.d
**11**	8.31 (8.16–8.46)	0.81 ± 0.05	Inactive		n.d
**12**	7.11 (6.97–7.24)	0.96 ± 0.02	6.84 (6.55–7.13)	0.73 ± 0.09	0.23 (0.06–0.41)
**13**	8.44 (8.13–8.75)	0.84 ± 0.03	8.36 (8.15–8.58)	0.37 ± 0.03	0.27 (0.20–0.34)

^a^ Values of potency and efficacy were obtained by forcing the E_max_ to 1. Inactive: compounds E_max_ < 0.10. n.d. bias factors could not be estimated.

**FIGURE 4:**
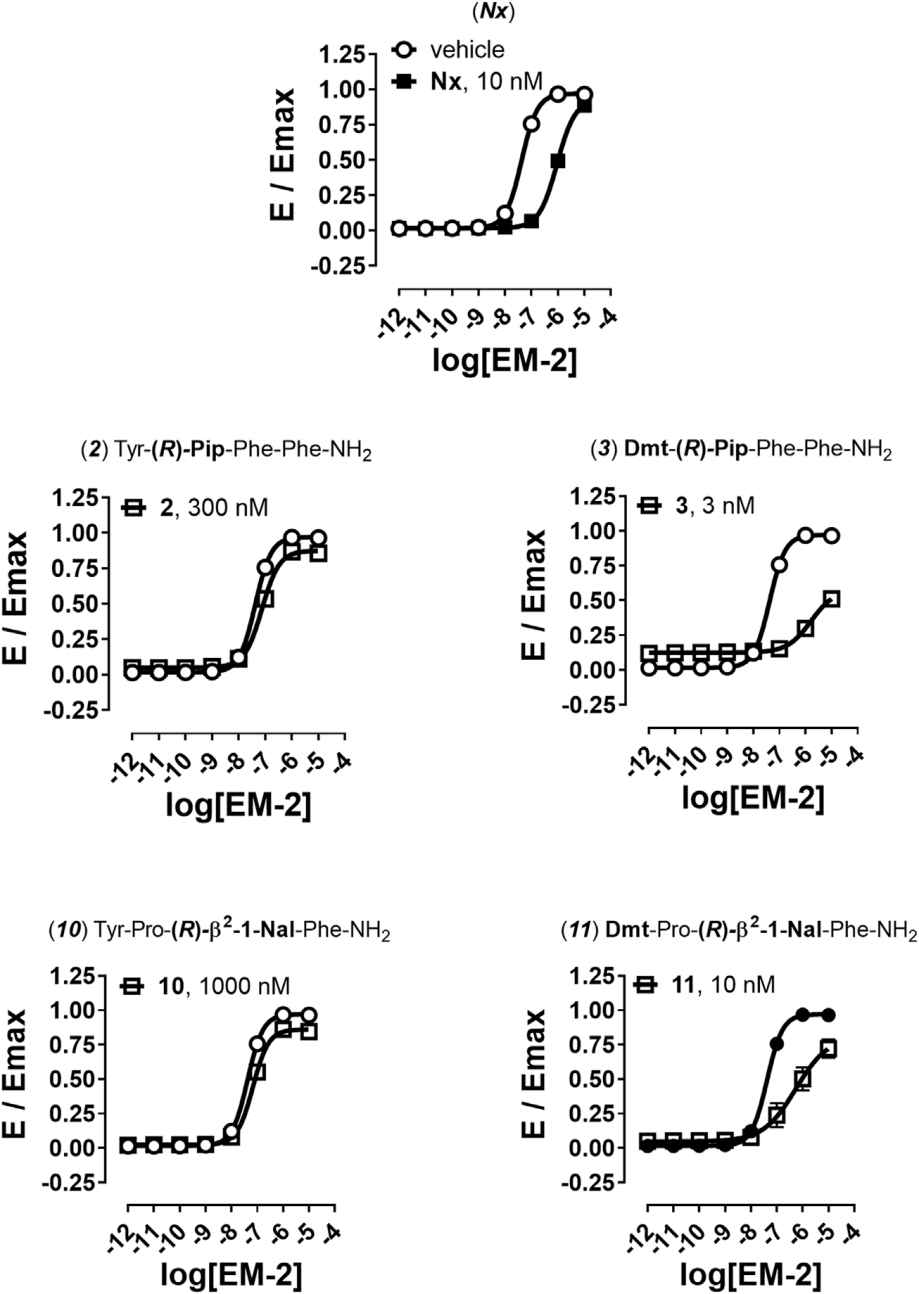
Antagonism experiments for β-arrestin 2 recruitment. Effects of Nx (10 nM), 2 (300 nM), 3 (3 nM), 10 (1,000 nM), and 11 (10 nM) in challenging EM-2 concentration-response curves in mu-β-arrestin 2 BRET interaction assay. Data are mean + sem of four independent experiments performed in duplicate.

Finally, to better understand the nature of action at the mu receptor for compounds displaying very weak (<0.10) efficacy for β-arrestin 2 recruitment, analogs **2**, **3**, **10**, and **11** were assayed in parallel to naloxone (**Nx**) in antagonism experiments. In these experiments a fixed concentration of antagonists challenged the concentration-response curve to EM-2 generating a rightward shift (Figure 4 **).**

Nx caused a shift to the right of EM-2 concentration-response curve with high potency value, without altering its maximal effect (Figure 4). High concentrations of compounds **2** and **10** generated a weak rightward shift of the concentration response curve to EM-2 with no modification of maximal effect and the obtained antagonist potencies were in the micromolar range (Figure 4). Conversely, the two Dmt derivatives **3** and **11**, tested at nanomolar concentrations, robustly shifted to the right the EM-2 concentration response curve, acting as potent mu receptor-β-arrestin antagonists (Figure 4). The results of antagonism experiments are summarized in Table 5.

**TABLE 5:** Antagonists parameters.

		pK_b_ (CL_95%_)
Nx	Naloxone	9.40 (9.23–9.57)
2	Tyr-*(R)-*Pip-Phe-Phe-NH_2_	6.77 (6.63–6.91)
3	Dmt-*(R)-*Pip-Phe-Phe-NH_2_	10.10 (9.95–10.24)
10	Tyr-Pro-*(R)-*β^2^-1-Nal-Phe-NH_2_	6.20 (5.96–6.43)
11	Dmt-Pro-*(R)-*β^2^-1-Nal-Phe-NH_2_	9.40 (9.12–9.67)

Values of antagonist potency for Nx and EM-2, analogs **2**, **3**, **10**, and **11** in the mu-β-arrestin 2 interaction assay.

## 4 Discussion

When designing new mu receptor agonists as potential analgesic drugs, it is necessary to take into consideration many factors that make a good candidate. In fact, such compounds should have high affinity, potency and selectivity for the mu receptor, be enzymatically stable and lipophilic enough to be able to cross the cellular membranes. In addition, according to biased agonism hypothesis they should selectively activate the G protein pathway downstream of the receptor (Grim et al., 2020). In the present manuscript we attempted to evaluate all these features.

According to the IUPAC definition, lipophilicity is the affinity of a molecule for a lipophilic environment and may help estimate its absorption, distribution, and transport through cellular membranes (Liu et al., 2011). The log*P* value is an important parameter for foreseeing the pharmacokinetic features of a novel compound (Arnott & Planey, 2012). Generally, higher lipophilicity provides better central nervous system (CNS) penetration. However, too high lipophilicity can result in the increased non-specific plasma protein binding and sequestration in fatty tissues (Arnott et al., 2013). Thus, compounds with moderate lipophilicity can most effectively penetrate the BBB (Gabathuler, 2010). The most effective intestinal absorption after oral administration was reported for drugs with log*P* value between 1.35–1.8 (Hansch et al., 1987). The highest uptake to the CNS is observed for compounds with lipophilicity log*P* values from 1.5 to 2.8, with the best BBB permeation for molecules with Log*P* around 2.1 (Hansch & Leo, 1979; Gabathuler, 2010; Gharagheizi et al., 2010).

Peptides are generally hydrophilic compounds, unable to cross the BBB by passive diffusion to reach the CNS in the amount sufficient to activate appropriate receptors. The inspection of the log*P* values reported in Table 1 revealed that analogs **1**–**11** fulfill the criterion of lipophilicity optimal for BBB penetration, as opposed to EM-2 with log*P* = 1.19. This low log*P* value is in agreement with the fact that EM-2 elicits a very strong analgesic effect only after direct administration to the CNS (Perlikowska and Janecka, 2014).

Due to degradation, physiological effects produced by opioid peptides are usually short-lasting. Appropriate chemical modifications of EMs result in obtaining more stable analogs. Introduction of D-amino acids or non-proteinogenic amino acids, including β-amino acids is a well-known strategy to increase peptide stability, since peptidases are unable to cleave amide bonds produced by such amino acids (Janecka et al., 2008). In the series of EM-2 analogs presented here, introduction of unnatural amino acids into positions 2 or 3 resulted in good enzymatic stability of the peptides. Clearly further *in vivo* studies are needed to establish if the reported chemical modifications are sufficient for generating EM-2 derivatives able to reach the central nervous system after peripheral administration and to promote long lasting effects.

All tested EM-2 analogs displayed very high mu opioid receptor binding affinity and selectivity. In the calcium mobilization assay they behaved as potent and selective mu receptor agonists. Therefore, these compounds were suitable for investigation of the distinct intracellular pathways related to mu opioid receptor activation.

With the BRET assay for mu receptor-transducer interaction, an already well-established readout for distinguishing G protein vs. β-arrestin coupling at several GPCRs, important pharmacological aspects of the developed analogs could be assessed (Malfacini et al., 2015; Piekielna-Ciesielska et al., 2018; Sturaro et al., 2022). Importantly, calcium mobilization and BRET mu-G protein interaction assays nicely correlated, in terms of both potency and efficacy (Supplementary Figure S6), thus confirming their value for investigating the pharmacological profile of novel ligands at the mu opioid receptor. The following rank order of potency was obtained in both tests:

9 ≥ 5 ≥ 3 ≥ 1 ≥ 13 ≥ 7 ≥ 11 ≥ 8 ≥ 4 ≥ EM-2 ≥ 2 ≥ 12 ≥ 10 ≥ 6

Interestingly, in all cases, introduction of Dmt in position 1 (odd numbered analogs) increased the potency of the parental analogs (even numbered analogs). As far as efficacy is concerned, this value for all analogs was comprised between 0.68 and 1.11, thus indicating that the chemical modifications had no major effects in terms of ligand efficacy for mu-G protein recruitment.

The mu-β-arrestin 2 interaction depicted instead a greater variability in terms of efficacy, from very weak/inactive (<0.1) to full agonists. Importantly, analogs **2**, **3**, **10**, and **11** showed intrinsic activities lower than 0.1 and were, therefore, tested for their capacity to antagonize the EM-2-induced mu-β-arrestin 2 coupling. Nx, the standard opioid antagonist, showed high value of potency in line with that reported in the literature (Rizzi et al., 2016; Costanzini et al., 2021). All these analogs displayed antagonist activity and, intriguingly, the potency of the two Dmt derivatives (**3** and **11**) was very high. As expected, the antagonist potency of the compounds in mu-β-arrestin 2 experiments was in good agreement with their agonist potency in mu-G protein studies. Interestingly enough, those compounds acting as antagonists of mu-β-arrestin 2 coupling displayed the lowest E_max_ values in mu-G protein interaction studies. This is in line with the findings obtained with other mu receptor ligands of different chemical structures; in fact, compounds such as oliceridine, PZM21, and SR-17018 that has very low (if any) efficacy in mu-β-arrestin 2 experiments also displayed reduced E_max_ in mu-G protein studies (Gillis et al., 2020b).

In terms of bias, by computing all G protein and β-arrestin 2 datasets with the approximated operational model for slopes not significantly different from one (see method section), we observed that only analogs four and five were truly unbiased, with compound **5** behaving as a very potent mu receptor unbiased agonist. The remaining eleven analogs displayed a different degree of bias towards G protein with the following rank order:

6 > 8 ≥ 7 ≥ 1 ≥ 9 ≥ 13 ≥ 12 ≥ EM−2=4=5

Very importantly, analogs **10**, **11**, **2**, and **3** were not included in the rank order above because their bias factors could not be finely estimated due to their lack of efficacy at recruiting β-arrestin 2. Nevertheless, a visual inspection of bias plots (Supplementary Figure S5) and comparative analysis of the results recommend the inclusion of the four compounds at the top of this list. Therefore, Phe^3^ to (*R*)-β^2^-Nal swap produced the highest G protein vs. β-arrestin 2 discrepancy, followed by Pro^2^ to (*R*)-Pip and to (*R*)-β^2^-Ala exchange.

It is very difficult to rationalize effector-specific structure-activity relationship since very subtle chemical modifications may promote vast effects. However, very recent structural evidence (Zhuang et al., 2022) suggests that balanced mu agonists such as fentanyl, morphine or DAMGO form interactions with both TM3 and TM6/7 sides of the mu receptor ligand binding pocked while G protein biased agonists such as PZM21, oliceridine, and SR17018 preferentially interact with the TM3 side. This evidence has been corroborated by the fact that mutations at the TM6/7 side of the binding pocket abolished β-arrestin recruitment and fentanyl analogues designed to reduce TM6/7 interactions displayed a G protein biased profile of action. Future molecular simulation studies performed with the mu receptor active structures reported (Zhuang et al., 2022) may establish if the potent and balanced mu agonist compound **13** and the potent G protein biased agonist compound **11** display a similar pattern of interaction with the TM3 and TM6/7 sides of the mu receptor ligand binding pocked.

Finally, as far as the biological significance of the mu receptor biased agonism is concerned, we would like to underline that discrepant results have been reported in the literature. In fact, as mentioned in the introduction, there is genetic and pharmacological evidence that G protein bias confers to mu receptor agonists a reduced side effect profile (Grim et al., 2020; Lambert and Calo’, 2020). However, there is also robust evidence that putative biased agonists are actually mu receptor partial agonists and this may be the reason for their safer profile (Gillis et al., 2020a; Azevedo Neto et al., 2020). Further studies are clearly needed to address this important and unresolved issue; for instance the *in vivo* evaluation and comparison of the analgesic vs. respiratory depressant and/or constipatory properties of the balanced mu agonist **13** and of the G protein biased agonist **11** may greatly contribute to this field.

In conclusion, with this study we offer the scientific community a nice toolbox of enzymatically stable, membrane penetrant, mu receptor selective agonists with different degrees of bias towards G protein. We foresee that these molecules may be useful in future studies for understanding the molecular basis of mu receptor biased agonism and its biological and possibly therapeutic significance and, on a longer term perspective, for facilitating the rational design of safer opioid analgesics.

## Data Availability

The original contributions presented in the study are included in the article/Supplementary Material, further inquiries can be directed to the corresponding author.
