# Supplementary material for: Functional selectivity of EM-2 analogs at the mu-opioid receptor
Source: Front Pharmacol. 2023 Feb 24;14:1133961. doi: 10.3389/fphar.2023.1133961 (PMC9998502; doi:10.3389/fphar.2023.1133961)
Supplement: Supplementary file 1 [file DataSheet1.pdf]

Supplementary Material

Functional selectivity of EM-2 analogs at the mu-opioid receptor

Justyna Piekielna-Ciesielska[1*#], Davide Malfacini[2#], Francine Medjiofack Djeujo[2], Chantal Marconato[2], Karol Wtorek[1], Girolamo Calo'[2], Anna Janecka[1].

# Equal contribution and first autorship

*** Correspondence:** Justyna Piekielna-Ciesielska, PhD: e-mail: justyna.piekielna@umed.lodz.pl

1 Table of Contents

**Figure S1**	LC-MS chromatograms of peptides 2-13	**page S-2-13**
**Figure S2**	Calcium mobilization experiments at delta receptor	**page S-14**
**Figure S3**	Calcium mobilization experiments at kappa receptor	**page S-15**
**Figure S4**	Effects of EM-2 and 1-13 on Rluc emitted light	**page S-16**
**Figure S5**	Bias plots for EM-2 derivatives	**page S-17**
**Figure S6**	Calcium vs. mu-G protein correlation	**page S-18**

Figure S1. LC-MS chromatograms of peptides 2-13

(2)

Tyr-(R)-Pip-Phe-Phe-NH2 MW~699.72 (585.7 · TFA)

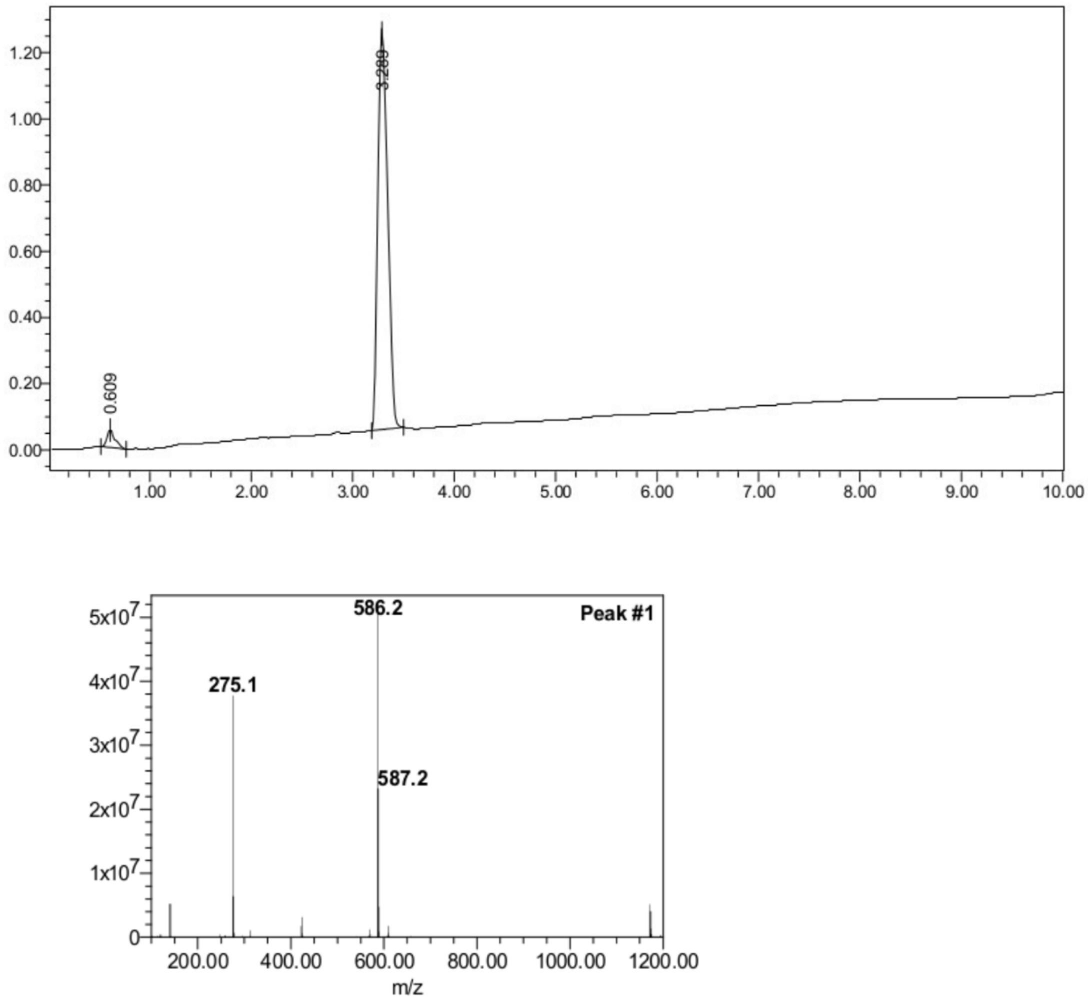

Retention Time 3.295 Channel Description
100.00-1200.00 ES+, Centroid, CV=40

(3)

Dmt-(R)-Pip-Phe-Phe-NH2 MW~727.77 (613.7 · TFA)

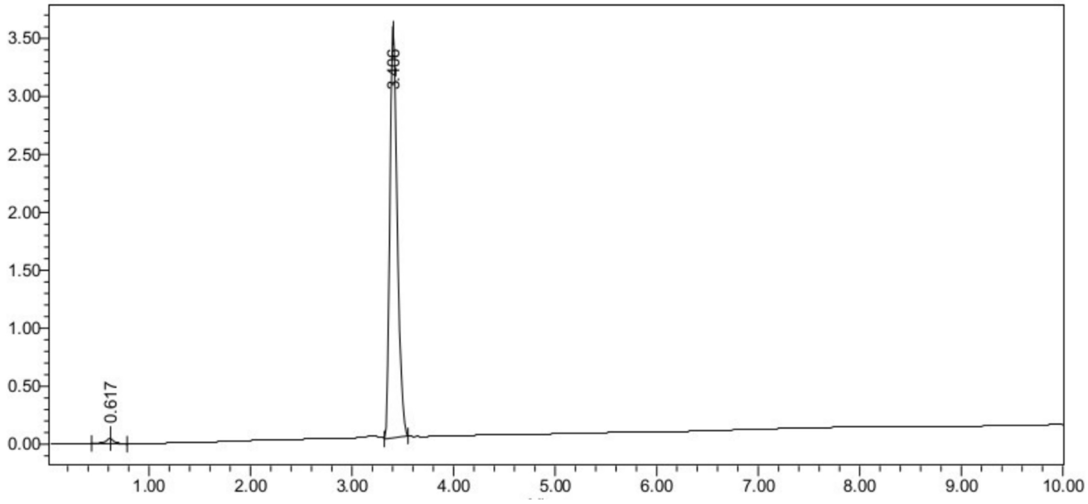

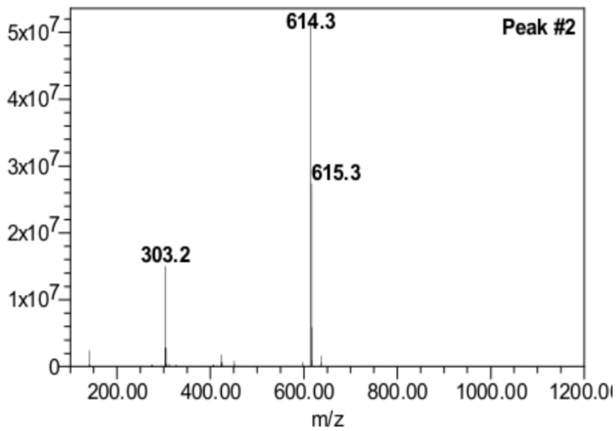

Retention Time 3.410 Channel Description
100.00-1200.00 ES+, Centroid, CV=40

(4)

Tyr-(R)-Nip-Phe-Phe-NH2   MW~699.72 (585.7 · TFA)

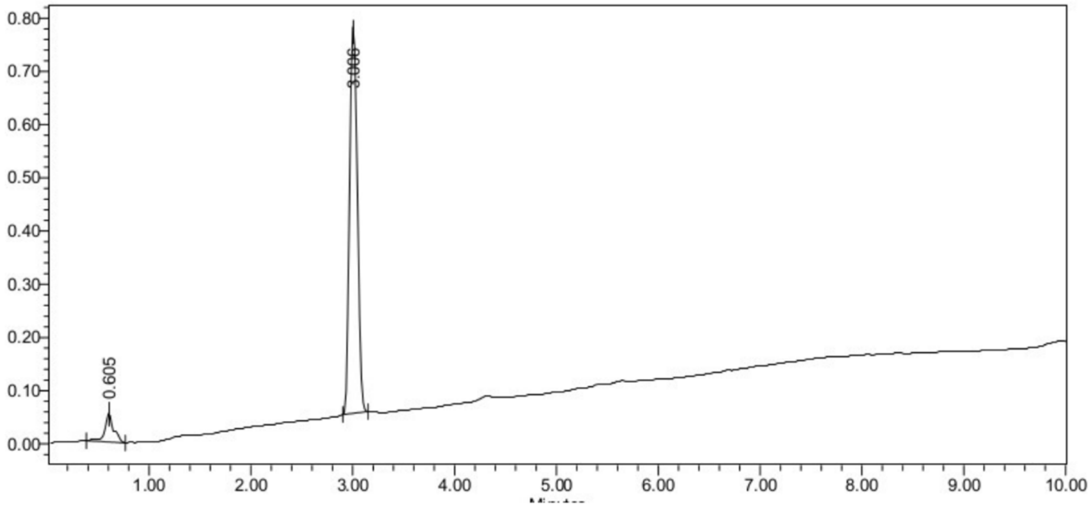

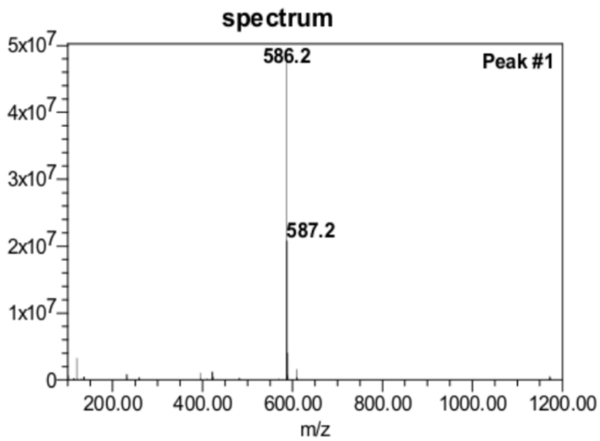

Retention Time 3.005 Channel Description
100.00-1200.00 ES+, Centroid, CV=40

(5)

Dmt-(R)-Nip-Phe-Phe-NH2 MW~727.77 (613.7 · TFA)

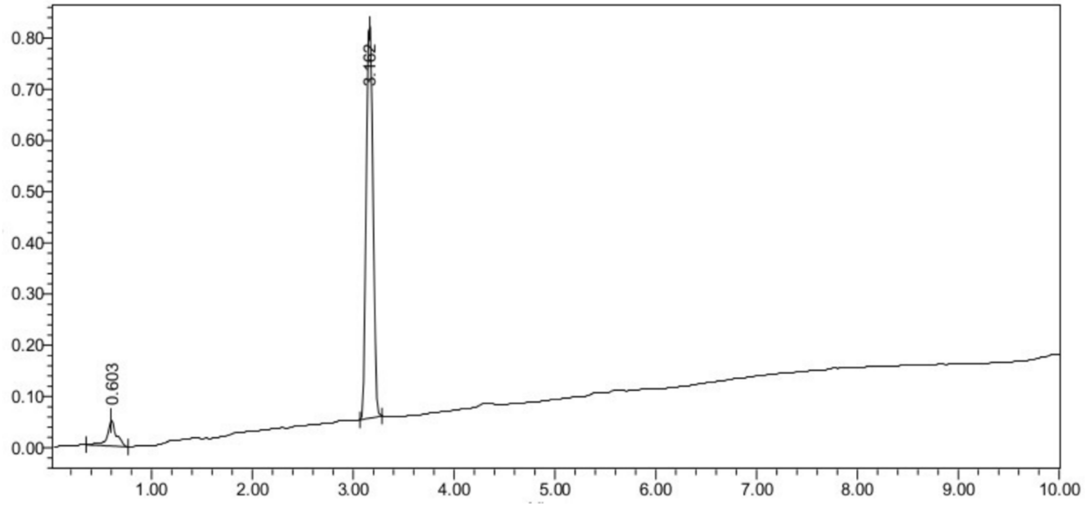

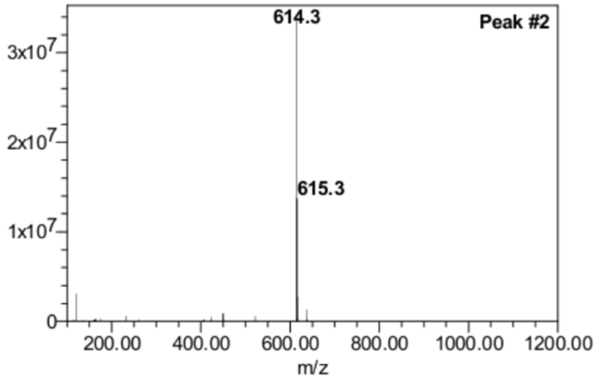

Retention Time 3.163 Channel Description
100.00-1200.00 ES+, Centroid, CV=40

(6)

Tyr-(R)-β2-Ala-Phe-Phe-NH2 MW~673.68 (559.7· TFA)

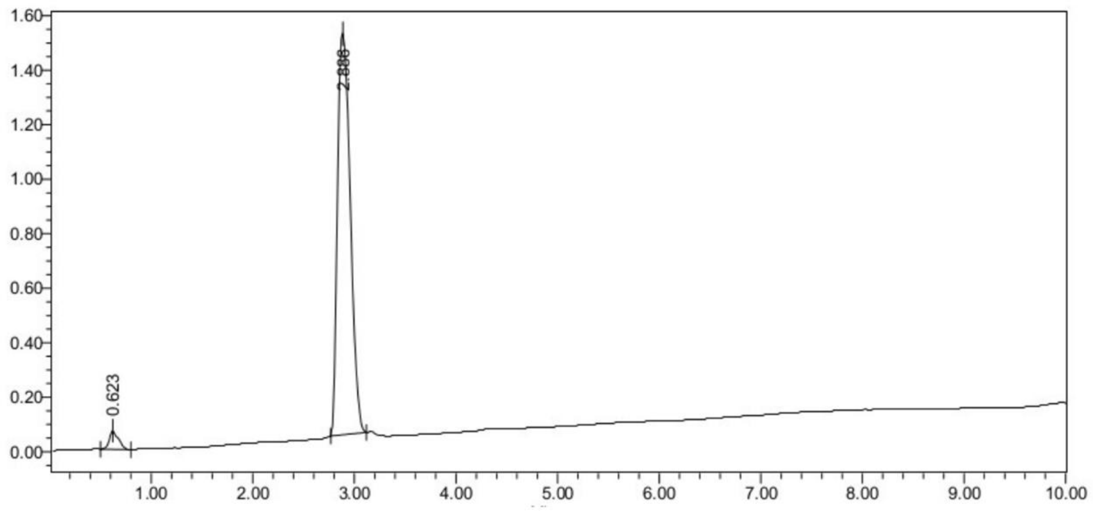

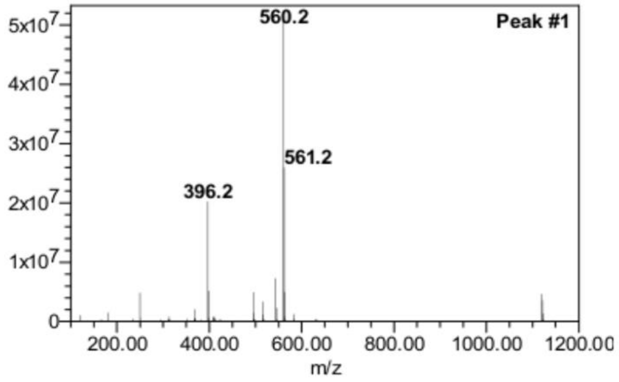

Retention Time 2.881 Channel Description
100.00-1200.00 ES+, Centroid, CV=40

(7)

Dmt-(R)-β2-Ala-Phe-Phe-NH2 MW~701.73 (587.7 · TFA)

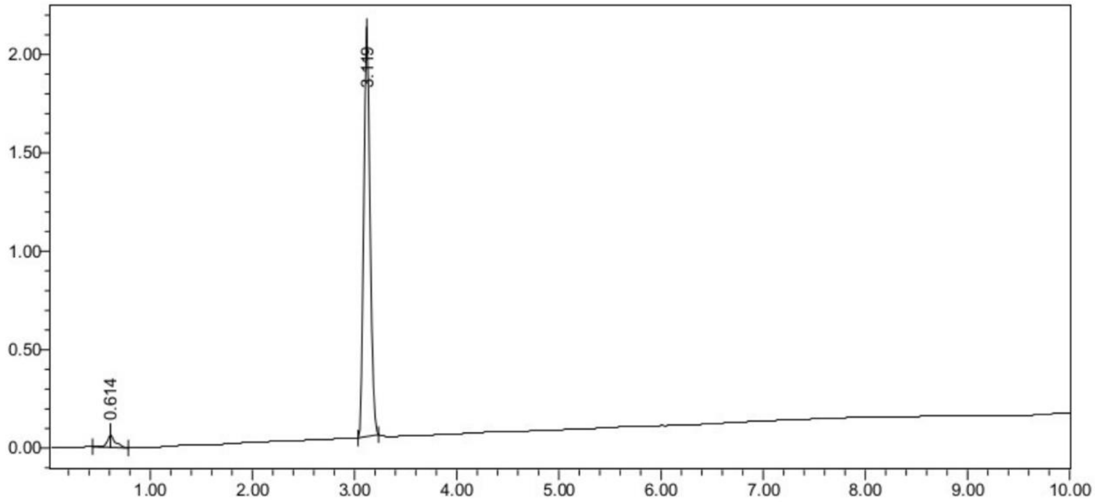

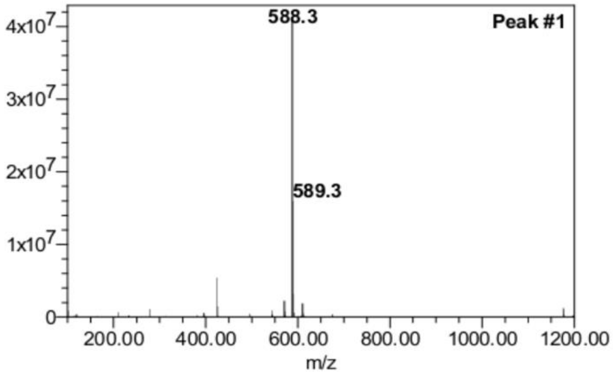

Retention Time 3.114 Channel Description
100.00-1200.00 ES+, Centroid, CV=40

(8)

Tyr-(R)-β3-Ala-Phe-Phe-NH2 MW~673.68 (559.7 · TFA)

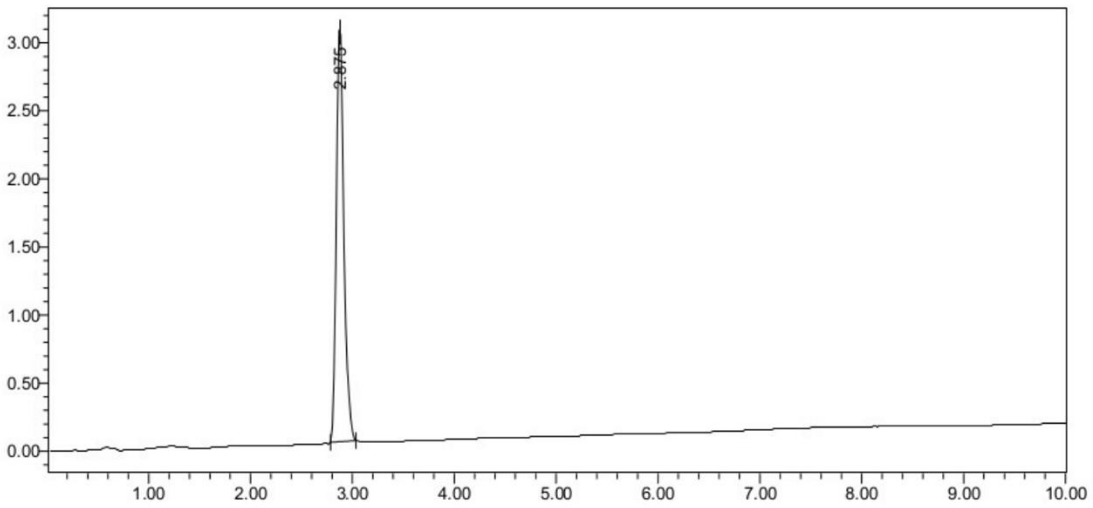

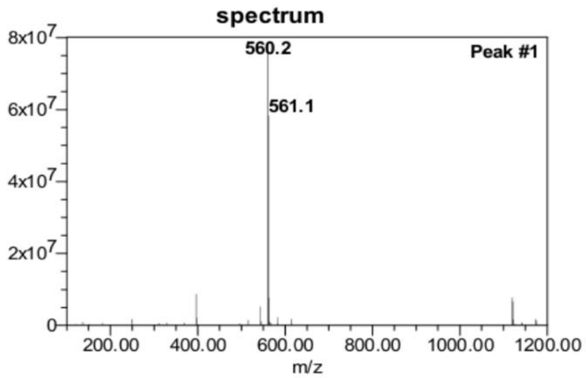

Retention Time 2.879 Channel Description
100.00-1200.00 ES+, Centroid, CV=40

(9)

Dmt-(R)-β3-Ala-Phe-Phe-NH2   MW~701.73 (587.7 · TFA)

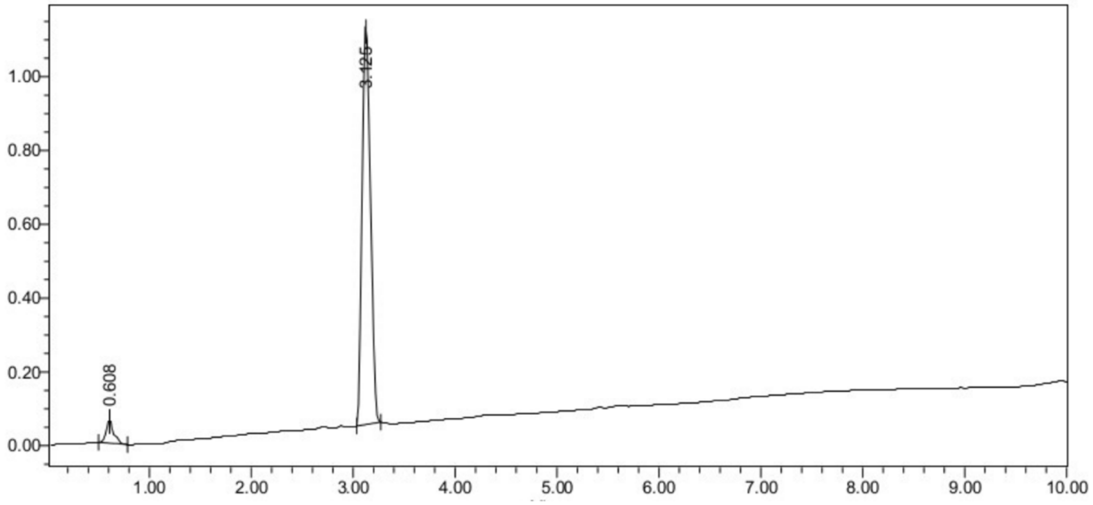

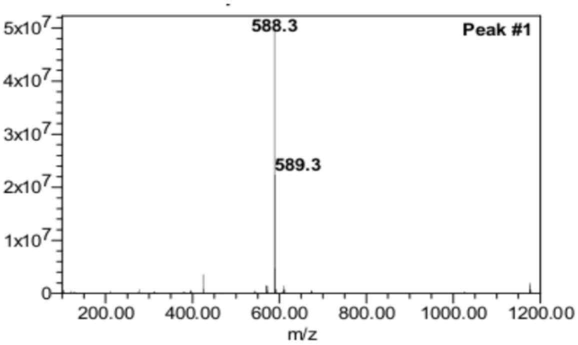

Retention Time 3.132 Channel Description
100.00-1200.00 ES+, Centroid, CV=40

(10)

Tyr-Pro-(R)-β2-1-Nal-Phe-NH2 MW~749.77 (635.8 · TFA)

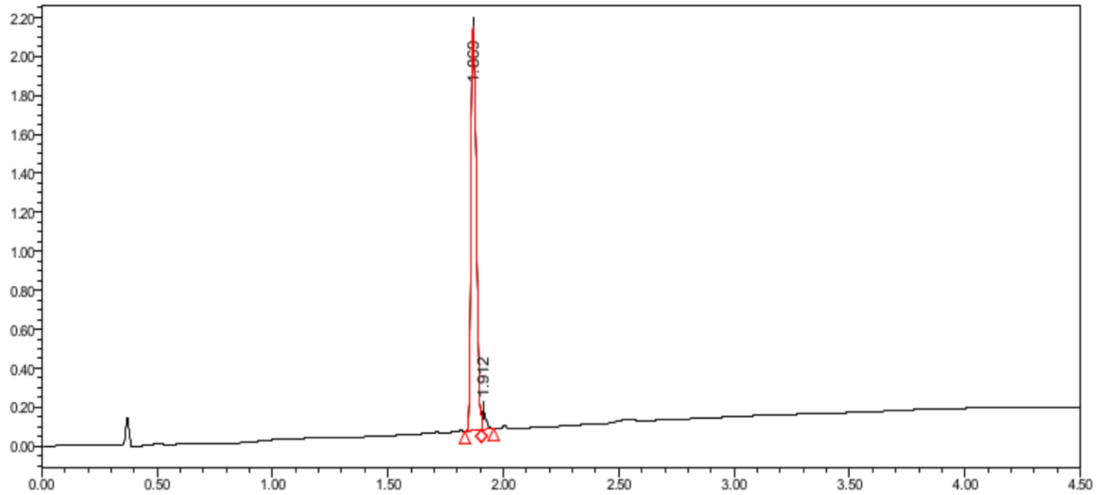

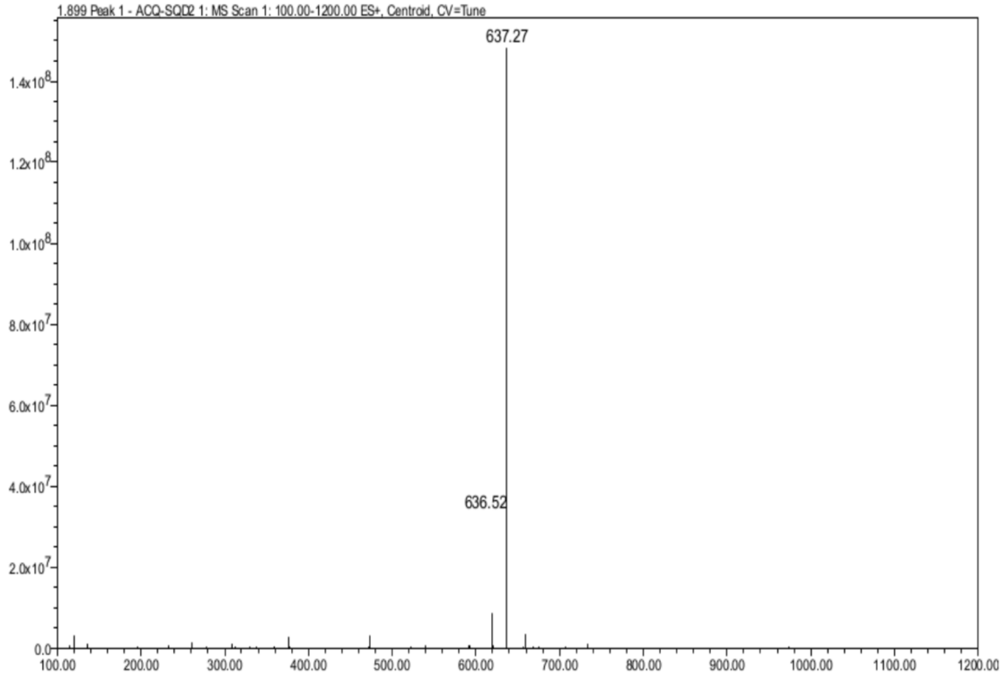

(11)

Dmt-Pro-(R)-β2-1-Nal-Phe-NH2 MW~777.83 (663.8 · TFA)

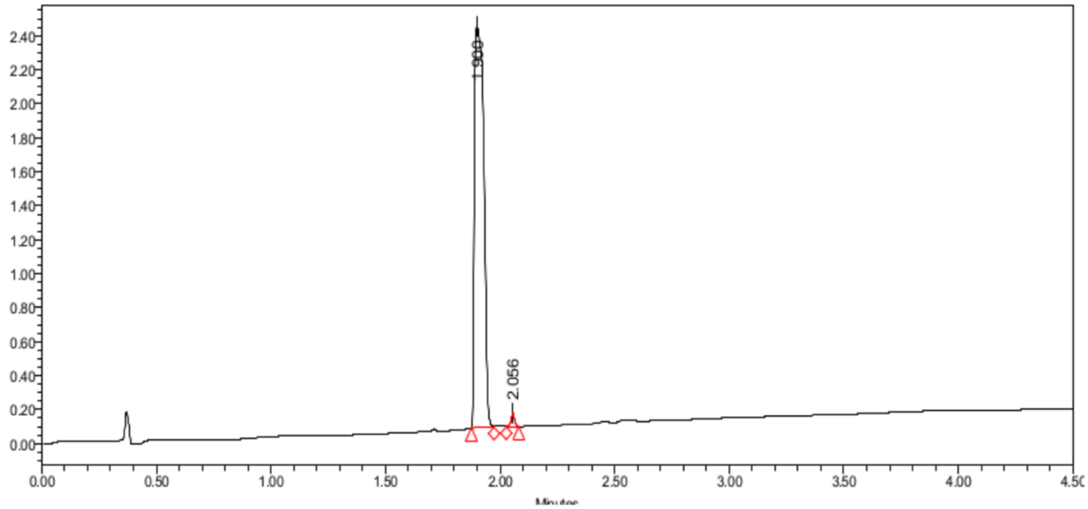

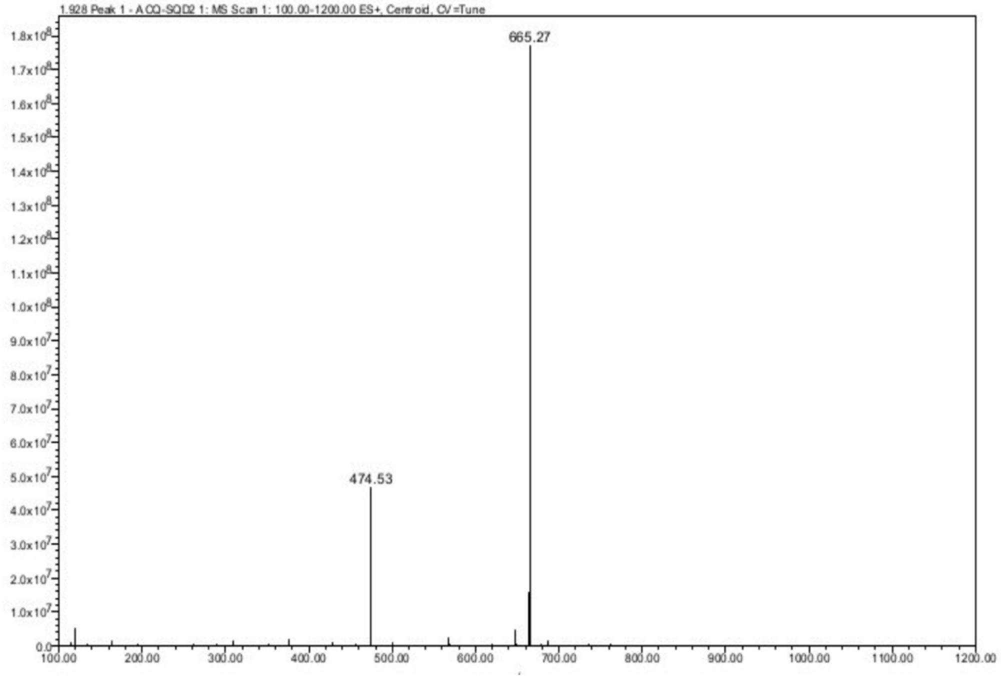

(12)

Tyr-Pro-(R)-β3-1-Nal-Phe-NH2 MW~749.77 (635.8 · TFA)

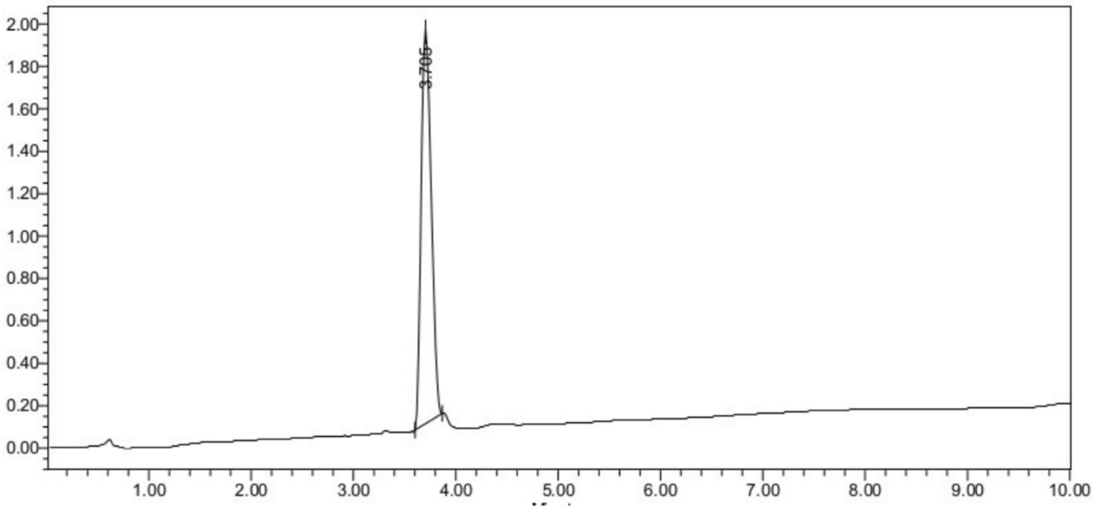

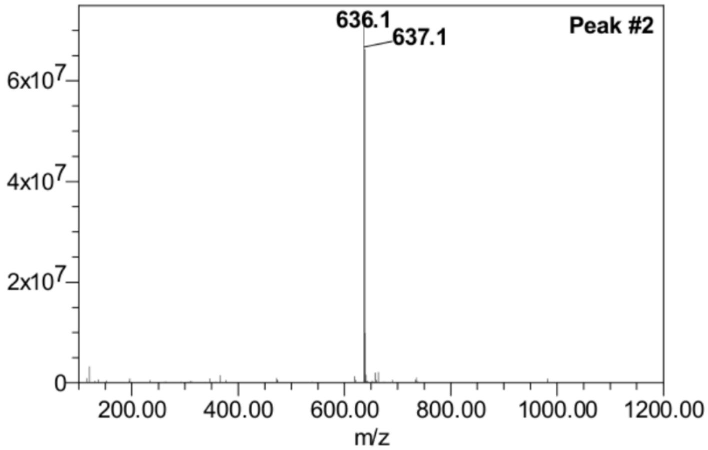

Retention Time 3.692 Channel Description
100.00-1200.00 ES+, Centroid, CV=40

(13)

Dmt-Pro-(R)-β3-1-Nal-Phe-NH2 MW~777.83 (663.8 · TFA)

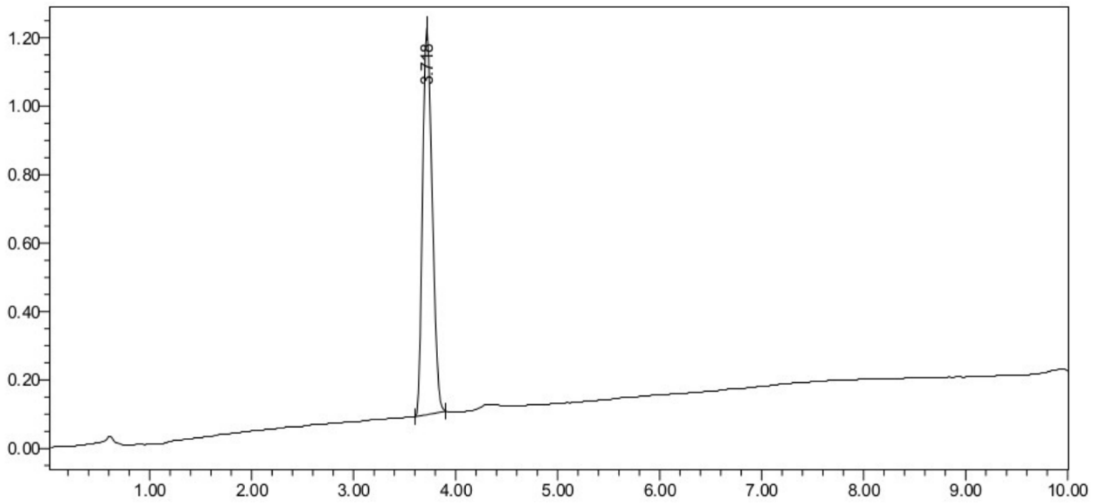

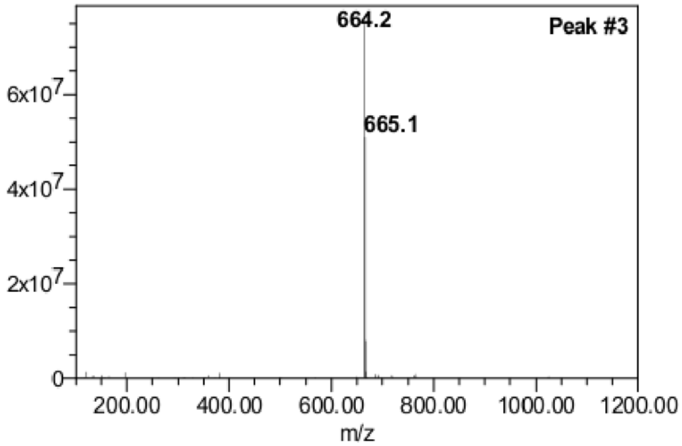

Retention Time 3.721 Channel Description
100.00-1200.00 ES+, Centroid, CV=40

Figure S2. Calcium mobilization experiments at delta receptor

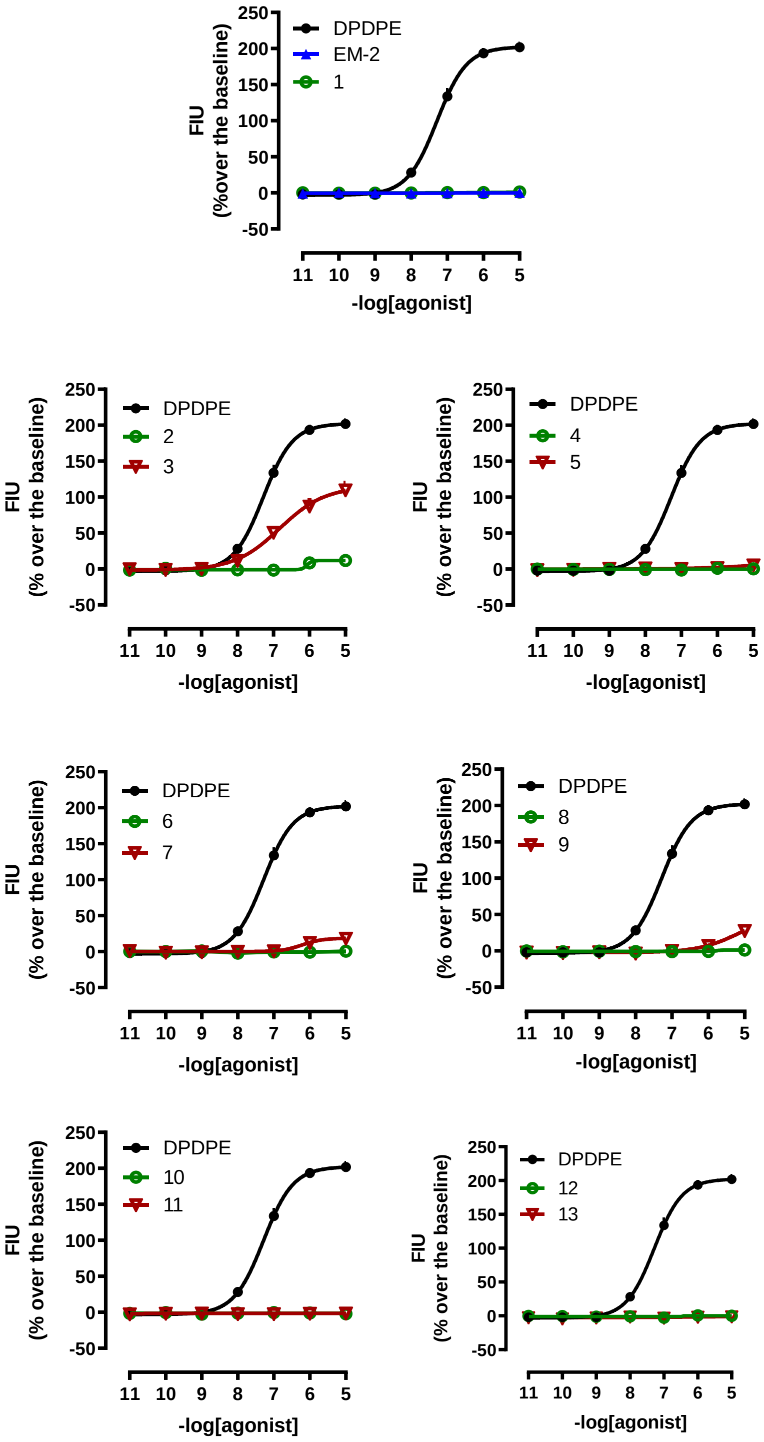

Concentration response curves to standard and tested compounds in calcium mobilization experiments performed in CHO$_{delta}$ cells stably expressing the G$\alpha_{qG66Di5}$ protein. Data are the mean ± SEM of at least 5 separate experiments performed in duplicate.

Figure S3. Calcium mobilization experiments at kappa receptor

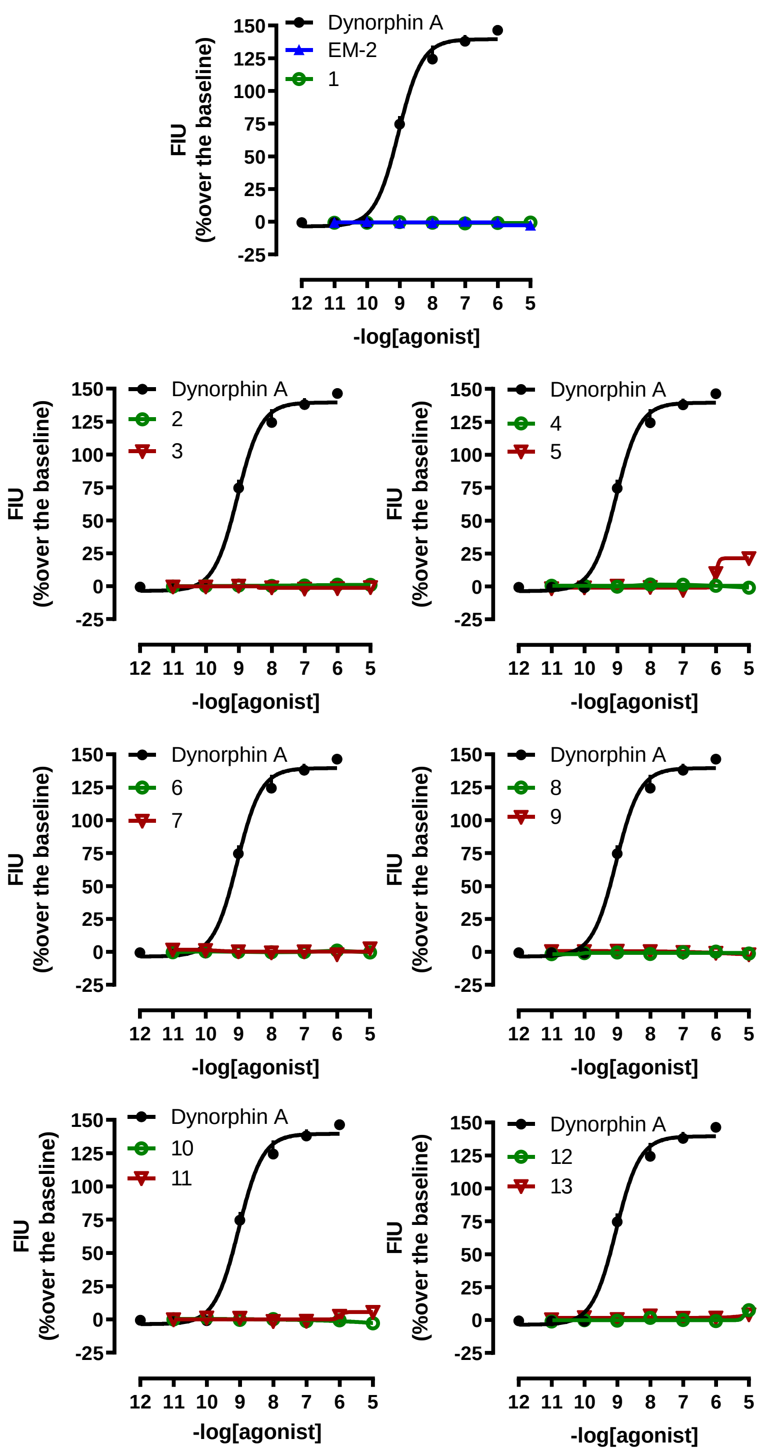

Concentration response curves to standard and tested compounds in calcium mobilization experiments performed in CHO_{kappa} cells stably expressing the $G\alpha_{qi5}$ protein. Data are the mean ± SEM of at least 5 separate experiments performed in duplicate.

Figure S4. Effects of EM-2 and 1-13 on Rluc emitted light

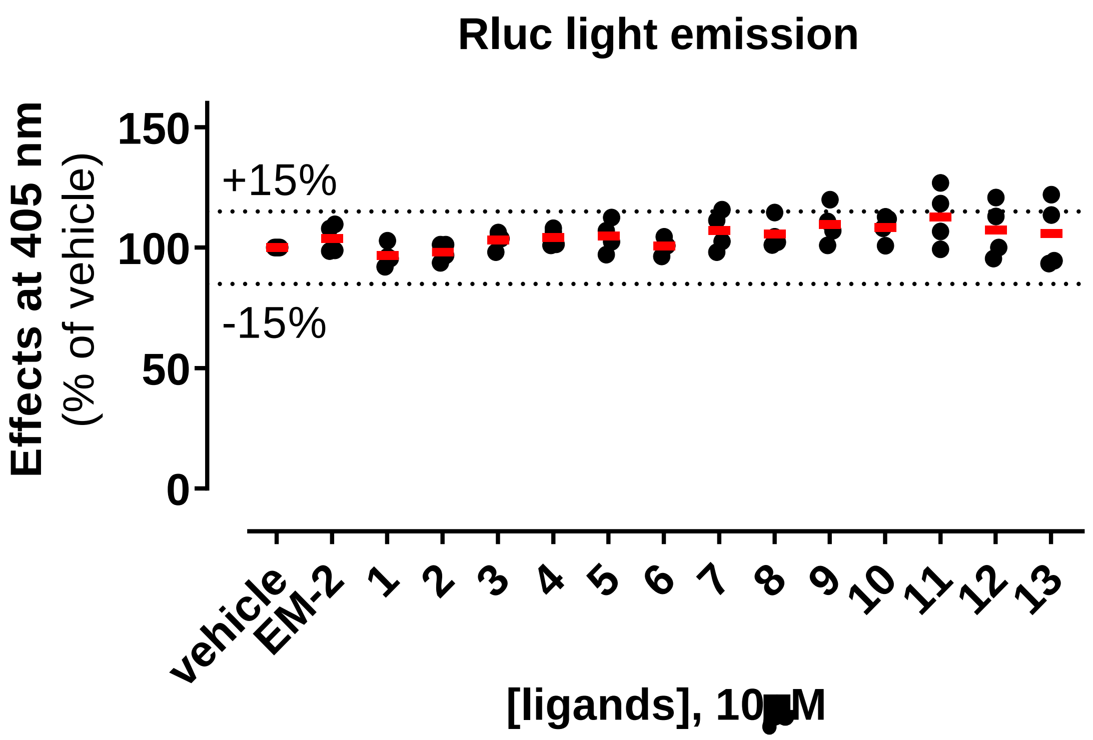

Effects of EM-2 and its thirteen derivatives on Rluc emitted light (averages are depicted as red line). Indicated are threshold for light alteration significance (dotted line). Data shown are mean of 4 independent experiments

Figure S5. Bias plots for EM-2 derivatives

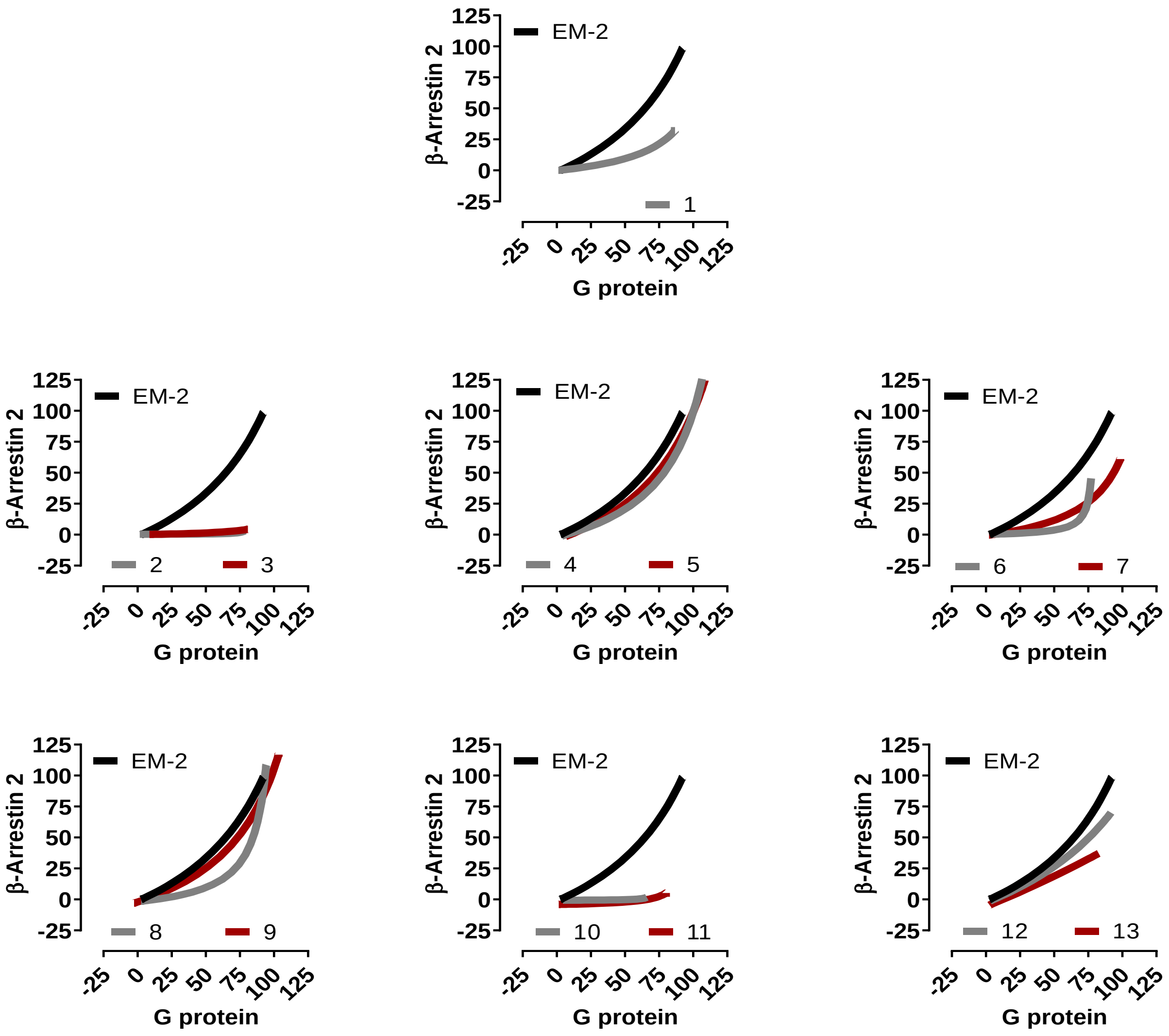

Bias plots for indicated ligands. EM-2 is black, Tyr^1-based derivatives are in dark red, and Dmt^1-based in dark grey. Bias plots represent the effects of each compound at equiactive concentrations as derived from the four parameters log equation.

Figure S6. Calcium vs. mu-G protein correlation

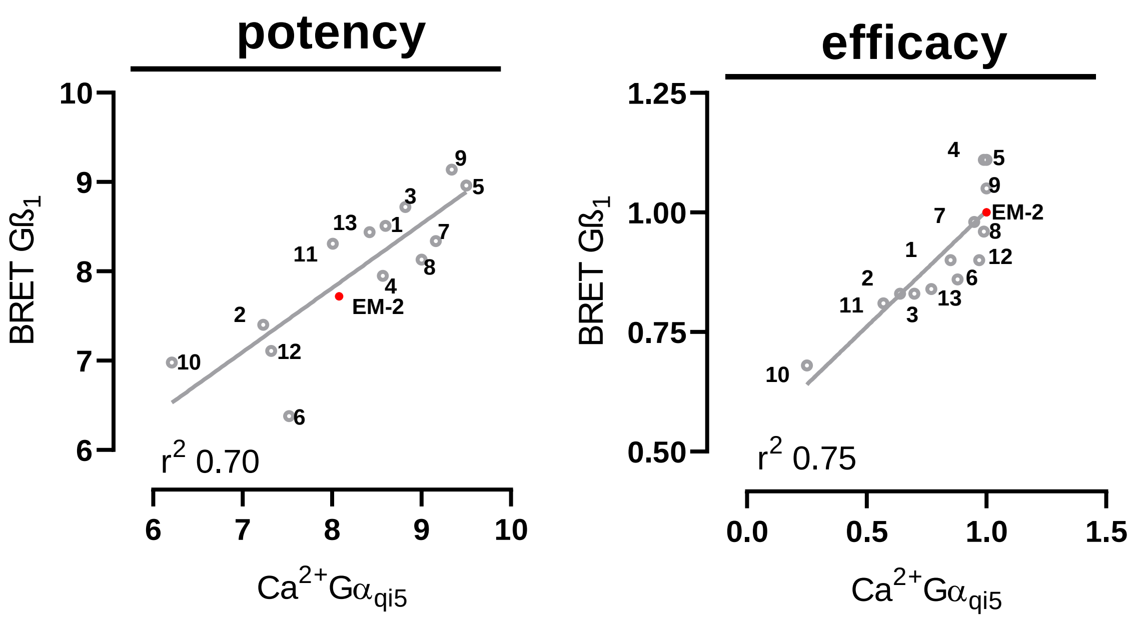

Comparison of potency and efficacy in the calcium mobilization (x-axis) and BRET mu-G protein interaction (y-axis) assays. Linear regression correlation coefficients are reported inside the graphs.
